# Origin and Functional Diversification of an Amphibian Defense Peptide Arsenal

**DOI:** 10.1371/journal.pgen.1003662

**Published:** 2013-08-01

**Authors:** Kim Roelants, Bryan G. Fry, Lumeng Ye, Benoit Stijlemans, Lea Brys, Philippe Kok, Elke Clynen, Liliane Schoofs, Pierre Cornelis, Franky Bossuyt

**Affiliations:** 1 Amphibian Evolution Lab, Biology Department, Vrije Universiteit Brussel, Brussels, Belgium; 2 Venom Evolution Laboratory, School of Biological Sciences, University of Queensland, St. Lucia, Queensland, Australia; 3 Department of Bioengineering Sciences, Research Group of Microbiology and VIB, Vrije Universiteit Brussel, Brussels, Belgium; 4 Unit of Cellular and Molecular Immunology, Vrije Universiteit Brussel, Brussels, Belgium; 5 Laboratory of Myeloid Cell Immunology, VIB, Brussels, Belgium; 6 Biomedical Research Institute, Hasselt University, Diepenbeek, Belgium; 7 Functional Genomics and Proteomics, Department of Biology, KU Leuven, Leuven, Belgium; Texas A&M University, United States of America

## Abstract

The skin secretion of many amphibians contains an arsenal of bioactive molecules, including hormone-like peptides (HLPs) acting as defense toxins against predators, and antimicrobial peptides (AMPs) providing protection against infectious microorganisms. Several amphibian taxa seem to have independently acquired the genes to produce skin-secreted peptide arsenals, but it remains unknown how these originated from a non-defensive ancestral gene and evolved diverse defense functions against predators and pathogens. We conducted transcriptome, genome, peptidome and phylogenetic analyses to chart the full gene repertoire underlying the defense peptide arsenal of the frog *Silurana tropicalis* and reconstruct its evolutionary history. Our study uncovers a cluster of 13 transcriptionally active genes, together encoding up to 19 peptides, including diverse HLP homologues and AMPs. This gene cluster arose from a duplicated gastrointestinal hormone gene that attained a HLP-like defense function after major remodeling of its promoter region. Instead, new defense functions, including antimicrobial activity, arose by mutation of the precursor proteins, resulting in the proteolytic processing of secondary peptides alongside the original ones.

Although gene duplication did not trigger functional innovation, it may have subsequently facilitated the convergent loss of the original function in multiple gene lineages (subfunctionalization), completing their transformation from HLP gene to AMP gene. The processing of multiple peptides from a single precursor entails a mechanism through which peptide-encoding genes may establish new functions without the need for gene duplication to avoid adaptive conflicts with older ones.

## Author Summary

Many amphibians defend themselves against predation and infections by secreting a mixture of gene-encoded toxins and antimicrobials. How does such an integrated defense weapon arise and how does it diversify to gain distinct antipredatory and antimicrobial functions? We took advantage of the availability of a sequenced genome for the African clawed frog *Silurana tropicalis* to provide the first comprehensive overview of an amphibian peptide defense arsenal, from its underlying genes to its bioactive components. A reconstruction of the evolutionary history of this gene repertoire allows us to elucidate the timing and mode of evolution of distinct defense functions. Our study shows that the basal transition from a gastrointestinal hormone function to a skin-secretory defense function was accompanied by major restructuring of regulatory sequences in the ancestral gene. Instead, subsequently diversifying defense genes underwent functional shifts by entering a bifunctional stage (by cleavage of two distinct defense peptides from a single precursor protein) and occasionally losing the original defense function (by loss of the original defense peptide). This pattern provides an evolutionary explanation for the processing of structurally or functionally unrelated toxins from the same or closely related precursor proteins in other poisonous and venomous animals.

## Introduction

In response to stress or injury, many amphibians release a viscous secretion through granular glands in their skin. In several frog families, the most abundant class of secreted molecules consists of peptides and proteins. Since the 1960s, several of these peptides have been identified as structural analogues of neurohormones that are evolutionarily conserved among vertebrates and play key roles in gastrointestinal functioning. These skin-secreted hormone-like peptides (hereafter abbreviated as HLPs) have been hypothesized to provide passive defense against predation, by disturbing gastrointestinal processes upon ingestion [1], [2], [3]. Other peptides however, were found to show little similarity to any vertebrate hormone, and their function was unclear at the time of their discovery [4], [5], [6].

In 1987, two 23-AA-long peptides in the skin secretion of the African clawed frog *Xenopus laevis* were shown capable to kill a broad range of microorganisms [7]. Both peptides, called magainins, were cleaved from a larger precursor protein as predicted from cloned cDNA sequences [7], [8] and are thus encoded by a single gene. The discovery of similar gene-encoded antimicrobial peptides (AMPs) in other amphibians fueled the perception that these animals possess a genetically controlled arsenal of antimicrobials in their skin that provides first-line protection against infectious microorganisms in their environment. AMPs are now considered key effectors of the innate immune system of many organisms, but amphibian skin secretions continue to be explored as promising sources of potential lead compounds for the development of new antibiotics. Amphibian species in which skin AMPS have been found typically secrete 5–20 different peptides [9], [10] although in *Odorrana* species, the number may exceptionally exceed even 100 [11]. A recent study suggested that AMPs in distantly related anuran lineages represent independently evolved defense arsenals [12]. However, the genetic mechanisms and processes that underlay the evolutionary origin and functional diversification of any single defense arsenal remains unknown.

Frogs of the family Pipidae (including the genera *Xenopus* and *Silurana*) possess some of the best-studied skin secretions of all amphibians [13]–[23]. The model species *X. laevis* for example, secretes the HLPs caerulein [24], levitide [25], and xenopsin [26] and apart from the two magainins, confirmed AMPs include PGLa [4], and pGQ [27], caerulein precursor factor (CPF), and two xenopsin precursor factors (XPF). cDNA sequences have shown that HLPs and AMPs in *X. laevis* are posttranslationally cleaved from strikingly similar precursor proteins [8], [28], and in some cases, both types of peptide are even processed from the very same precursor [e.g. the HLP xenopsin and the AMP xenopsin precursor fragment, the HLP caerulein and the AMP caerulein precursor fragment [5], [25], [29]. Consequently, these peptides are not only subject to the same gene-regulatory mechanisms, they also share interdependent evolutionary histories.

Previoulsy, we identified a precursor gene in the species *Silurana tropicalis* with homology to the caerulein genes of *X. laevis* [30]. Phylogenetic analyses showed that they represented a single gene lineage, which evolved from the *cholecystokinin* (*cck*) hormone gene in a pipid ancestor. In the present study, we combined transcriptome, genome, and peptidome data to obtain a comprehensive overview of the entire defense peptide arsenal that evolved from this gene lineage, from the underlying genes to the active peptides. Besides identifying new peptides with potential therapeutic applications, our analyses disclose the full gene repertoire that encodes the AMP/HLP arsenal of *S. tropicalis*, elucidates the birth-and-death process by which it diversified, and illustrate how peptides with new functions arose in the expanding arsenal. Throughout this study, we apply the nomenclature introduced by Conlon et al. [13]–[23] to describe new peptides, and extend its use to their precursor proteins and genes.

## Results

### 1. Charting the AMP/HLP gene repertoire of *Silurana tropicalis*

**Figure 1:**
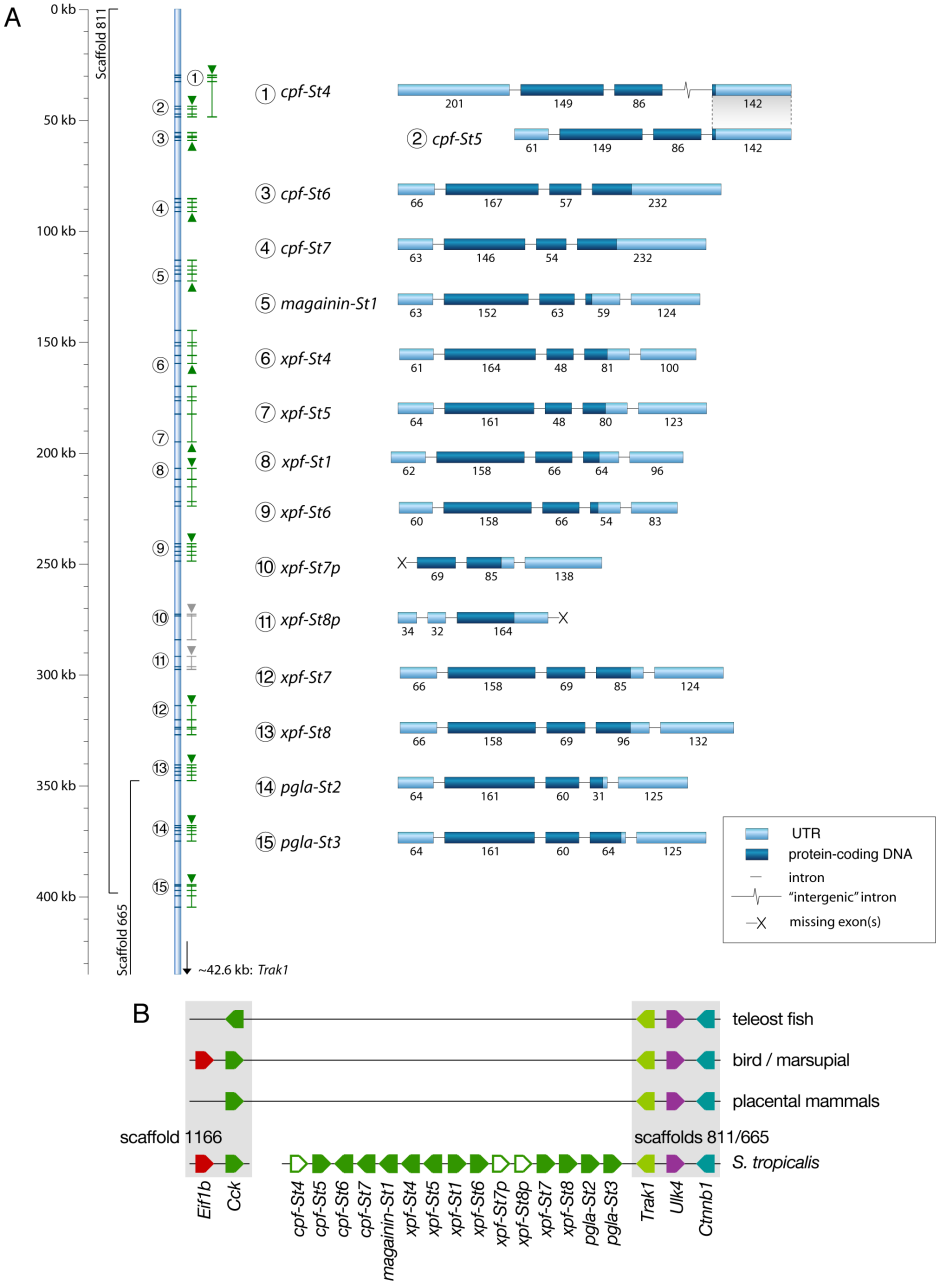
Genomic organization of the *Silurana tropicalis* AMP gene repertoire. (A) Gene cluster map showing gene and transcript positions (indicated by numbers 1–15 on the left) and gene orientation (indicated by upward or downward pointing triangles). Genes with incomplete coding sequences are colored grey. Exon organization of each gene/transcript is shown on the right (labeled by the numbers of the gene map), with exon lengths indicated as numbers below bars, untranslated regions colored light blue and coding sequences colored dark-blue. (B) Schematic representation of the *S. tropicalis* AMP gene cluster and adjacent genes showing preserved synteny with the *cck* gene in other vertebrates.

To chart the full repertoire of AMP genes in the *S. tropicalis* genome, we obtained new transcriptome data by preparing a cDNA library of skin samples of two *S. tropicalis* frogs. Comparative alignments and BLAST analyses of 384 randomly sequenced clones yielded 113 query sequences with homology to *X. laevis* mRNA transcripts and genes encoding known AMPs and HLPs (Table 1). This number corresponds to a remarkably high proportion (∼29%) of the recovered skin transcriptome. Together, they represent nine different mRNA transcripts, one of which encodes the recently described Xt6LP precursor [30] (Following the nomenclature of Conlon et al., this gene is hereafter called *cpf-St7*, where ‘*cpf*’ stands for ‘caerulein precursor fragment’, ‘*St*’ stands for *S. tropicalis* and ‘*7*’ indicates that it encodes the seventh CPF peptide known for this species). BLAST-screening of the *S. tropicalis* genome using the recovered transcripts identifies a single cluster of 15 homologous genes spanning a ∼380-kb region over the scaffolds 665 and 811 (Fig. 1A). They occur in both orientations, are composed of three (*cpf-St4*), four (*cpf-St5*, *cpf-St6*, and *cpf-St7*) or five exons (*magainin-St1*, *xpf-St4*, *xpf-St5*, *xpf-St6*, *xpf-St7*, xpf*-St8*, *pgla-St2*, and *pgla-St3*) and show considerable variation in length, ranging from ∼3.6 kb (*cpf-St6*) to over 23.5 kb (*xpf-St5*). One gene (*xpf-St8p*) shows signs of pseudogenization, with a degraded 5′-UTR and a premature stop-codon, while another (*xpf-St7p*) seems to lack a 5′-UTR and start codon altogether. The thirteen others are transcriptionally active, as evidenced by the nine query transcripts and by non-annotated EST sequences available in GenBank (Table 1; Text S1). One of these genes, *cpf-St4*, lacks a terminal exon but 17 GenBank EST sequences show that it is involved in the production of alternative splicing variants, by combining the *cpf-St4* exons with the terminal exon of the adjacent *cpf-St5* (Fig. 1A). No related genes were found on any other scaffold, suggesting that the entire AMP gene repertoire of *S. tropicalis* is organized in a single cluster. This cluster is flanked by the genes *trak-1*, *ulk-4* and *ctnnb-1* on scaffold 665, which in other vertebrates lie adjacent to *cck* (Fig. 1B), delivering solid genomic support for the hypothesis that the pipid AMP/HLP gene family evolved from an ancestral *cck* gene [30].

**Table 1:** Transcript abundance of *S. tropicalis* AMP genes in our cDNA library and in the EST division of GenBank.

Gene	Number of cDNA clones	Number of GenBank ESTs
*cpf-St4*	0	17
*cpf-St5*	15	24
*cpf-St6*	0	2
*cpf-St7*	29	21
*magainin-St1*	19	31
*xpf-St4*	10	9
*xpf-St5*	4	0
*xpf-St1*	0	0
*xpf-St6*	5	0
*xpf-St7p*	0	0
*xpf-St8p*	0	0
*xpf-St7*	7	6
*xpf-St8*	0	17
*pgla-St2*	22	10
*pgla-St3*	2	0

### 2. The AMP/HLP arsenal of *S. tropicalis*

**Figure 2:**
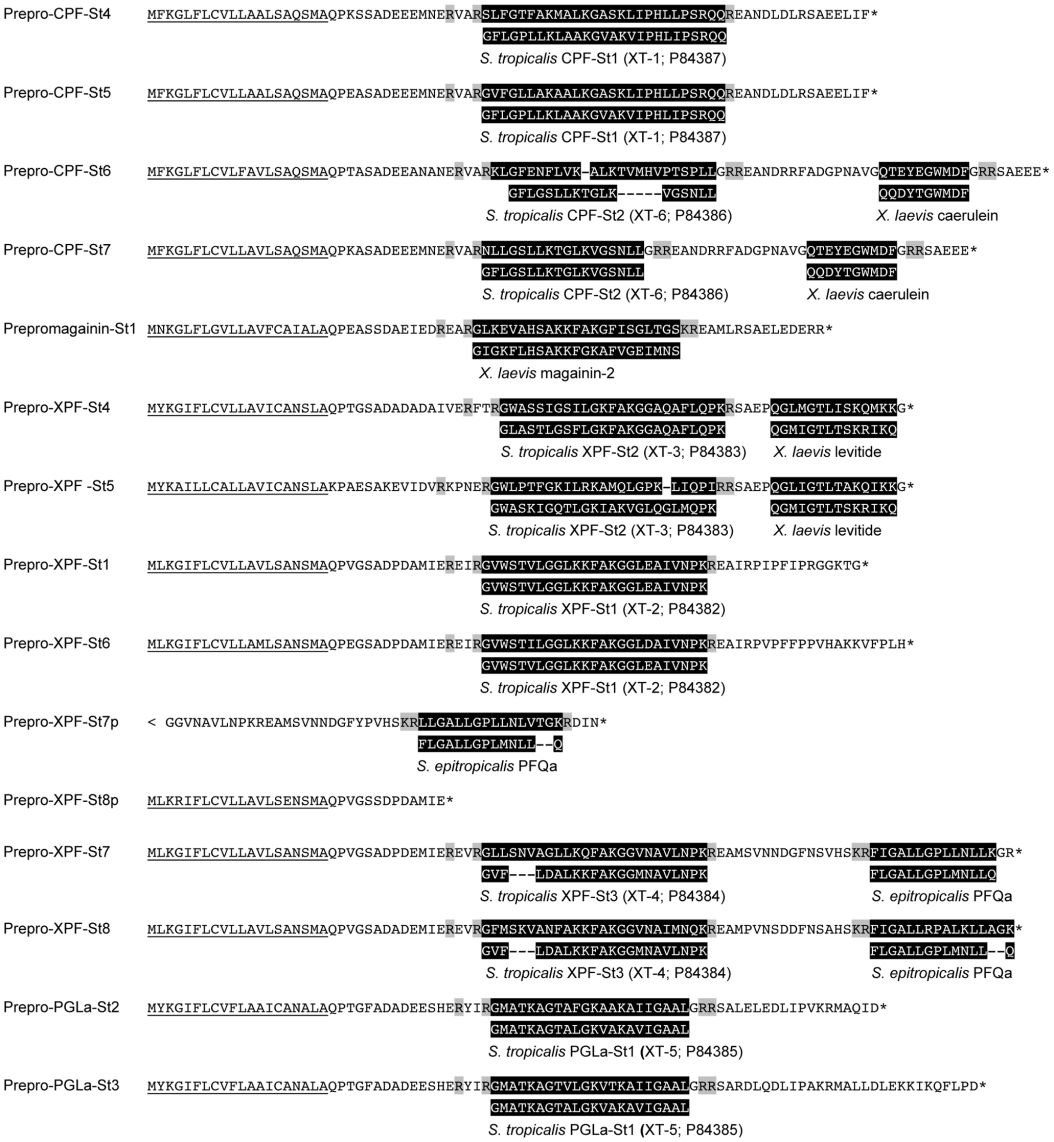
Precursors encoded by the *Silurana tropicalis* AMP gene repertoire. Peptides are predicted based on sequence homology to previously identified peptides (both highlighted in black) and putative cleavage sites (highlighted in gray). Underlined sequences represent predicted signal peptides. Asterisks represent translation stops.

All *S. tropicalis* AMP genes encode precursor proteins of 75 to 96 amino acids (AA) spanning exons 2–4 and containing an N-terminal signal peptide as predicted by the SignalP server [31]. Comparison of the inferred precursor proteins with previously isolated peptides from *Silurana* and *Xenopus* species allows us to predict the position and cleavage of functional peptides (Fig. 2). All precursor sequences share a region divided over exons 2 and 3, with sequence homology to previously reported AMPs. In nearly all cases, this region is flanked by an N-terminal -RXXR- motif and a C-terminal -RXXR-, -KR-, or -RR- motif, corresponding to common cleavage sites in other vertebrate peptide precursors. In addition, several of the *S. tropicalis* genes, similar to those of *X. laevis*, seem to encode secondary peptides, some of which show homology to known HLPs. The C-terminal region of prepro-CPF-St6 shows sequence similarity to the HLP caerulein, as previously reported for prepro-CPF-St7 [30]. Prepro-XPF-St4 and prepro-XPF-St5 share a C-terminal region with similarity to the *X. laevis* peptide levitide [25], and prepro-XPF-St7, prepro-XPF-St7p and prepro-XPF-St8 show similarity to peptide phenylalanine-glutamine-amide (pFQa), a 14-AA-long peptide of *Silurana epitropicalis* [16].

Nano-liquid chromatography tandem mass spectrometry data (nanoLC-MS/MS) from skin extracts confirm that *S. tropicalis* synthesizes a peptide arsenal that parallels the AMP/HLP diversity of *X. laevis*. Screening of these spectra against a database composed of AMP/HLP precursor proteins predicted from cDNA and gene sequences confirms the cleavage and posttranslational processing of 11 predicted peptides, while providing support (albeit with insignificant identity scores) for two more (Table 2, Text S2). These include nine of the predicted AMP homologues, two levitide-related peptides (Levitide-St1 and Levitide-St2) and a single homologue of pFQa (pFQa-St1). Our analysis additionally indicates the processing of a secondary peptide (PLD-St1) from prepro-PGLa-St3 without any apparent homology to known peptides. Visual inspection of this precursor confirms the proximity of an N-terminal -KR- site (Fig. 2). This cleavage site is separated from PLD-St1 by the dipeptide Met-Ala, indicating that a cleaved 16-AA-long peptide may be further processed by exopeptidases to obtain PLD-St1. Posttranslational modifications of individual residues are observed in six of the cleaved peptides, all of which undergo C-terminal amidation, and two of which undergo N-terminal pyroglutamate formation (Table 2).

**Table 2:** Overview of transcriptionally active *S. tropicalis* AMP genes, predicted peptides and their molecular weights.

Gene	Confirmed transcription	Predicted Peptide	Sequence^3^	Confirmed by nanoLC-MS/MS	Confirmed by other studies	M.W.
*cpf-St4*	yes^1^	CPF-St4	SLFGTFAKMALKGASKLIPHLLPSRQQ	yes	no	2938,66
*cpf-St5*	yes	CPF-St5	GVFGLLAKAALKGASKLIPHLLPSRQQ	yes	no	2812,68
*cpf-St6*	yes^1^	CPF-St6	KLGFENFLVKALKTVMHVPTSPLL_a_	no	no	2680,55
		Caerulein-St1	_p_QTEY_S_EGWMDF_a_	no	no	1365,55
*cpf-St7*	yes	CPF-St7	NLLGSLLKTGLKVGSNLL_a_	yes	no	1838,14
		Caerulein-St1	_p_QTEY_S_EGWMDF_a_	no	no	1365,55
*magainin-St1*	yes	Magainin-St1	GLKEVAHSAKKFAKGFISGLTGS	yes	no	2332,29
*xpf -St4*	yes	XPF-St4	GWASSIGSILGKFAKGGAQAFLQPK	yes	no	2518,37
		Levitide-St1	_p_QGLMGTLISKQMKK_a_	yes	no	1543,86
*xpf-St5*	yes	XPF-St5	GWLPTFGKILRKAMQLGPKLIQPI	no	no	2704,60
		Levitide-St2	_p_QGLIGTLTAKQIKK_a_	(yes)^4^	no	1479,91
*xpf-St1*	yes^2^	XPF-St1	GVWSTVLGGLKKFAKGGLEAIVNPK	no	yes	2568,48
*xpf-St6*	yes	XPF-St6	GVWSTILGGLKKFAKGGLDAIVNPK	yes	no	2568,48
*xpf-St7*	yes	XPF-St7	GLLSNVAGLLKQFAKGGVNAVLNPK	yes	no	2507,46
		PFQa-St1	FIGALLGPLLNLLK_a_	(yes)^4^	no	1479,95
*xpf-St8*	yes^1^	XPF-St8	GFMSKVANFAKKFAKGGVNAIMNQK	no	no	2685,42
		PFQa-St2	FIGALLRPALKLLAGK	no	no	1680,08
*pgla-St2*	yes	PGLa-St2	GMATKAGTAFGKAAKAIIGAAL_a_	yes	no	2018,10
*pgla-St3*	yes	PGLa-St3	GMATKAGTVLGKVTKAIIGAAL_a_	yes	no	2069,24
		PLD-St1	LLDLEKKIKQFLPD	yes	no	1698,99

1 , by GenBank ESTs.

2 , by previously reported peptides.

3 , posttranslational modifications:a, C-terminal amidation; p, pyroglutamate formation; S, tyrosine sulfation.

4 , predicted but with insignificance scores (<26).

M.W., molecular weight.

### 3. Structural and functional analysis of *S. tropicalis* AMPs

The majority of the predicted peptides show the typical structural features of amphibian AMPs (Table 3). Most of them have the potential to form an alpha-helix according to secondary structure predictions [32], are cationic at a pH of 7.0, and show an alternated sequence of hydrophylic/cationic and hydrophobic AAs, which in an alpha-helical configuration results in a strong amphipathic structure. Upon contact with cell membranes, the cationic side of the helix allows interaction with negatively charged phospholipid heads on the membrane surfaces, while the hydrophobic side facilitates alignment with, or intrusion into the intermembrane region, potentially inducing membrane pore formation and eventually, cell lysis [33], [34].

**Table 3:** Physicochemical properties of *S. tropicalis* AMPs and HLPs.

		Predicted helicity^1^				
Peptide	Length (AA)	Absolute (AA)	Relative (%)	Net Charge^2^	Hydrophobic moment^3^	Hydrophobicity^4^
CPF-St4	27	16	59,3	4,1	2,34	47,0
CPF-St5	27	14	51,9	4,1	1,94	41,5
CPF-St6	25	15	60,0	4,1	1,69	35,5
CPF-St7	18	12	66,7	4	3,84	45,2
magainin-St1	23	17	73,9	3,1	2,94	37,4
XPF-St4	24	13	54,2	3	2,61	47,9
levitide-St1	14	11	78,6	4	2,09	31,6
XPF-St5	24	12	50,0	4	4,1	35,5
levitide-St2	14	9	64,3	4	1,57	35,0
XPF-St1	25	14	56,0	3	3,02	36,6
XPF-St6	25	14	56,0	3	3,16	36,5
XPF-St7	25	14	56,0	3	2,61	37,1
PFQa-St1	14	12	85,7	3	4,36	55,4
XPF-St8	25	22	88,0	5	2,5	35,6
PFQa-St2	16	13	81,3	3	2,85	44,5
PGLa-St2	22	18	81,8	5	2,1	42,6
PGLa-St3	22	16	72,7	5	2,06	39,2
PLD-St1	14	9	64,3	0	4,15	n.a.^5^

1 predicted by the PsiPred server (Buchan et al 2010).

2 at pH = 7.0.

3 based on the combined consensus scale of Tossi et al. (2002).

4 expressed as % TFA/AcN solvent at HPLC elution time.

5 not available.

It was recently argued that AMPs, besides being effectors of innate immune response, may play a role in antipredator defense through their cytolytic effects [35]. We therefore investigated the activity of 17 peptides by concentration-dependent assays in liquid media against both microorganisms and vertebrate cells. Target cells included two gram-negative bacteria (*Escherichia coli* and *Pseudomonas aeruginosa*), two gram-positive bacteria (*Staphylococcus aureus* and *Micrococcus luteus*), a fungus (*Saccharomyces cerevisiae*), a protozoan parasite (*Trypanosoma brucei*), mouse (*Mus musculus*) red blood cells, and mouse spleen cultures (containing a mixture of T-lymphocytes, macrophages and myeloid cells). The variable nature of these cells required the use of different measures of peptide activity (see Materials and Methods) but combined, their results support a number of distinct patterns.

First, 13 of the 17 synthesized peptides show antimicrobial activity as evidenced by minimum inhibitory concentrations (MIC). These include 12 peptides homologous to known AMPs, and PFQa-St2 (Table 4). In all cases examined, subculturing from MIC solutions to agar media lacking peptides did not yield any colonies, confirming that growth of the population was inhibited by effectively killing the cells.

**Table 4:** Activity of *S. tropicalis* peptides against a broad range of cell types.

	Gram-negative bacteria		Gram-positive bacteria		Eukaryote microorganisms		vertebrate cells	
	*E. coli*	*P. aeruginosa*	*S. aureus*	*M. luteus*	*S. cerevisiae*	*T. brucei*	*Erythrocytes*	*T-lymphocytes*
Peptide	MIC (µM)	MIC (µM)	MIC (µM)	MIC (µM)	MIC (µM)	LC_95_ (µM)	HC_50_ (µM)	IC_50_ (µM)
CPF-St4	2–4	4–8	1	0,5	16	2–4	64	2
CPF-St5	1	4–8	1	0,5	16	1–2	64	8
CPF-St6	16	>256	>256	4	64	16–256	256	32–64
CPF-St7	128	>256	32	2	64	2–64	256	128
magainin-St1	64	>256	>256	32	128	8–>256	>256	>256
XPF-St4	32	128	>256	0,5	64	4	256	64
XPF-St5	1–2	8–16	16	1	32	2–4	128	8–16
XPF-St1	4	32	32–64	0,5	32	2–64	128	16
XPF-St6	2–4	16	8–16	0,5	8	2–4	64	8
XPF-St7	8	32	128	1	32	2–64	>256	16
XPF-St8	1	4	128	0,5	8	1–8	64	16
PFQa-St2	32	256	16	4	128	16–32	256	128
PGLa-St2	16	128	>256	2	64	16–32	>256	64

MIC, minimum inhibitory concentration, the lowest peptide concentration (in µM) in a series of twofold dilutions at which no growth was detected; LC_95_, the lowest peptide concentration at which at east 95% of parasites was killed after 30 minutes; HC_50_, the lowest peptide concentration causing at least 50% hemolysis; IC_50_, the lowest peptide concentration causing at least 50% inhibition of Concanavalin A-induced T-cell proliferation. In case of variation among repetitive tests, observed minima and maxima for these values are given, respectively.

Second, ten of the peptides are to some extent capable of inducing lysis of red blood cells. Observed hemolytic activity is generally relatively low, with peptide concentrations inducing 50% hemolysis (HC_50_) ranging down to 64 µM. In addition, 12 peptides are capable to inhibit the Concanavalin A-stimulated proliferation of T-lymphocytes in spleen cell cultures. Peptide concentrations inducing 50% inhibition of T-cell proliferation (IC_50_) range down to 2 µM (CPF-St4) while higher concentrations typically eliminate any proliferation. Concanavalin A induces T-cell proliferation indirectly, by stimulating macrophages to activate T-cells. Consequently, the peptides may inhibit proliferation directly (by suppression or lysis of T-cells), indirectly (by inhibition or lysis of macrophages), or both. These results confirm that amphibian AMPs may indeed contribute to antipredator defense through cytolytic and/or immunosuppressive effects.

Third, the tested peptides show notable variation in activity against any single cell type. For *S. aureus* for example, MIC values range from >512 µM (e.g., magainin-St1) down to 1 µM (e.g., CPF-St5). For *T. brucei*, a single peptide may additionally show variation in activity across repeated assays. This pattern may be related to the use of pleomorphic parasite cultures, representing a variable mixture of two parasite forms (“long-slender” and “short-stumpy”) with potentially different susceptibility to membrane permeabilization.

Fourth, our assays do not provide evidence of target cell specificity or complementary target spectra among different peptides as observed in other frogs (e.g., [36]). Although there is substantial variation in the sensitivity of cell types to the AMPs, (e.g, the gram-positive *M. luteus* is highly sensitive to most peptides), the measured activities of peptides across cell types are correlated. In other words, peptides either seem effective against the broad range of cell types (e.g., CPF-St5) or hardly effective at all (e.g., magainin-St1). Previous studies have demonstrated that relatively weak AMPs from the same amphibian species may yield an increased antimicrobial effect when applied in combination [11], [36], [37], [38]. We searched for the existence of such synergistic effects among the *S. tropicalis* AMPs by MIC assays against *S. aureus* in which peptides were pairwise combined in a 1∶1 stochiometry. Our analyses reveal a single case of synergistic activity: combination of two of the weakest AMPs, magainin-St1 and PGLa-St1, yields a combined MIC of 64 µM (32 µM for each peptide), while individually, magainin-St1 had no apparent effect on *S. aureus* (MIC>512 µM) and PGLa-St1 had a very weak effect (MIC = 512 µM).

### 4. Evolutionary diversification of the pipid AMP gene repertoire

#### Origin of the pipid AMP gene promoter

Similar to CCK, AMPs have been found in the gastronintestinal tract of *X. laevis* [27]. However, the transition of the neurohormone function of CCK to the defense function of these AMPs implies that changes in gene regulatory mechanisms entailed a shift in gene expression to the skin glands. The transcriptional regulation of CCK is well-studied and comparative analyses of its promoter region have shown the evolutionary conservation of several cis-regulatory elements (CREs) across vertebrates [39], [40]. These include a TATA-box, GC-box, one or several E-box elements (consensus sequence CANNTG), and a combined cAMP response element/tetradecanoylphorbol-13-acetate response element (CRE/TRE; consensus sequence TGCGTCAG). Binding of basic Leucine-zipper (bZIP) transcription factors like CREB and Jun/Fos (AP-1) heterodimers to the CRE/TRE responsive element has been shown to increase *cck* promoter activity. Instead, binding of basic-helix-loop-helix (bHLH) transcription factors like the c-Myc/Max heterodimer to the E-box responsive element inhibits CRE/TRE activation, indicating a mechanism of negative cooperativity between the juxtaposed elements [40], [41].

**Figure 3:**
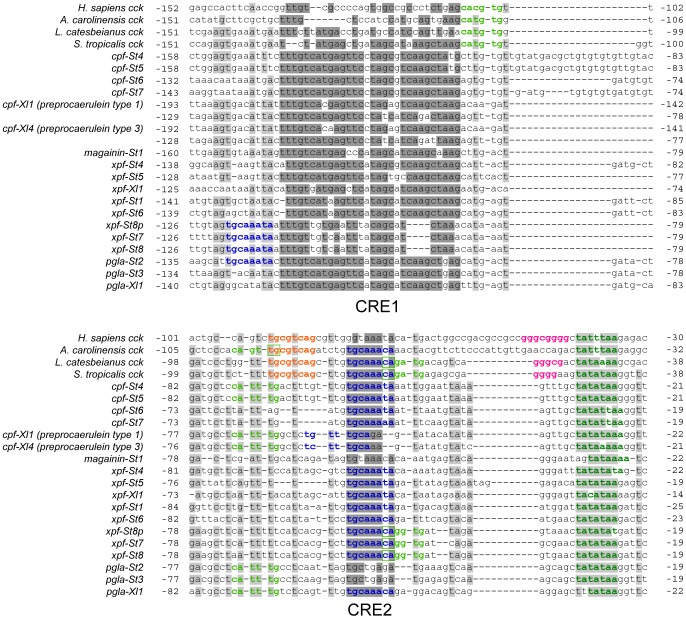
Comparative alignment of the promoter regions of vertebrate *cck* genes and pipid AMP genes. Sites with >75% sequence conservation across AMP gene promoters are indicated in grey; those additionally discussed as conserved sequence motifs in the text are indicated in dark grey. Sequence motifs corresponding to known or putative regulatory elements are colored as follows: dark green, TATA-box; light green, E-box; Orange, CRE/TRE element; pink, GC-box; dark blue, CRE2-conserved octamer.

Comparative alignment of the promoter regions of the pipid AMP genes with those of tetrapod *cck* genes shows a major renovation of otherwise evolutionary conserved sequence motifs in the promoter region (Fig. 3). The TATA-box is retained in all AMP genes, but the E-box and CRE/TRE elements, conserved at a fixed positions 10–20 bp apart in *cck* gene promoters, are absent, implying loss of the abovementioned negative cooperativity mechanism. These binding sites became evolutionarily degraded to give way to high sequence variation across AMP genes (Fig. 3). Instead, our analyses identify two motifs that show strong evolutionary conservation across AMP gene promoters. The first motif (CRE1) is 36–41 bp long and starts approximately 120–155 bp upstream of the transcription initiation site. Motif discovery searches using MEME [42] show that CRE1 occurs twice within the promoter region of the *X. laevis* caerulein genes. In addition, the same analyses recognize homologous sequences in the *cck* core promoters of both *Lithobates catesbeianus* (American bullfrog) and *S. tropicalis* as variants of the same motif, suggesting that the evolution of CRE1 reached an identifiable stage at the time of divergence of ranid and xenopodine frogs, during early anuran radiation. The second motif (CRE2) is located approximately 50 bp upstream of the TATA-box and contains the conserved octamer TGCAAANA in 13 out of 16 investigated AMP genes in *S. tropicalis* and *X. laevis*. This motif evolved to an inverted orientation in the *X. laevis* caerulein gene promoters, and originated secondarily in a more upstream position in the promoter regions of *xpf-St7* through *pgla-St2*. The TGCAAANA octamer is present at the same position in the *cck* gene promoters of *Anolis carolinensis* (anole lizard), *L. catesbeianus*, and *S. tropicalis*, indicating that it may represent a much older, previously overlooked CRE retained in the AMP gene promoters. The level of sequence conservation of the two motifs, and the fact that they occur (sometimes twice) in the majority of AMP gene promoters is a strong indication that they are subject to purifying selection, and play a fundamental role in the genetic regulation of the AMP/HLP arsenal. In addition, the presence of both motifs in *cck* genes of various taxa implies that their evolution predated the loss of *cck*-specific CREs and that their evolution was well underway before the diversification of pipid AMP genes.

Screening of the two newly identified promoter motifs against the TRANSFAC public database [43] predicts the potential binding of several other transcription factors known to be involved in innate immunity. The CRE1 motif in the majority of AMP genes contains two sites with sequence similarity to known binding elements of the bZIP transcription factors C-Fos, C-Jun, Fra-1, JunB, JunD, and XBP1. Several of those have been shown to play a role in various immune responses [44], including the expression of AMP genes in other vertebrates [45]. In addition, CRE2 shows strong similarity to known binding elements of CCAAT/Enhancer binding proteins (C/EBP) and POU class 2 homeobox factor 1 (POU2F1), again transcription factors associated with the synthesis of antimicrobial peptides and proteins in mammals [46]. Binding of these transcription factors to overlapping sites has been observed before in the promoter of *interleukin-8*, where C/EBP causes activation of the gene while POU2F1 causes repression [46].

The pipid AMP gene promoters show little similarity to promoters characterized in skin-expressed AMP genes of other amphibians. Analysis of the *gaegurein 4* gene in *Rana rugosa* revealed the lack of a TATA box, and the presence of Dl and NF-IL6 transcription factor binding sites, known to regulate acute phase immune response in mammals and insects [47]. The promoter regions of the *bombinin-like peptide 3* (*blp3*) and *bombinin-like peptide 7* (*blp7*) genes in *Bombina orientalis* [48], besides NF-IL6 elements, contain target sites for NF-kB, another key regulator of inflammatory and immune responses. While the NF-IL6 binding motif in *blp3* shows resemblance to CRE2 in the AMP gene promoters (NF-IL6 is a member of the C/EBP protein family), dl and NF-kB binding sites are absent in the AMP gene promoters. These differences in core promoter motifs indicate that AMP arsenals in distantly related frog lineages are not only encoded by independently evolved gene families [12], but are also differently “wired” into the innate immune response pathway.

#### Phylogeny of the pipid AMP gene repertoire

**Figure 4:**
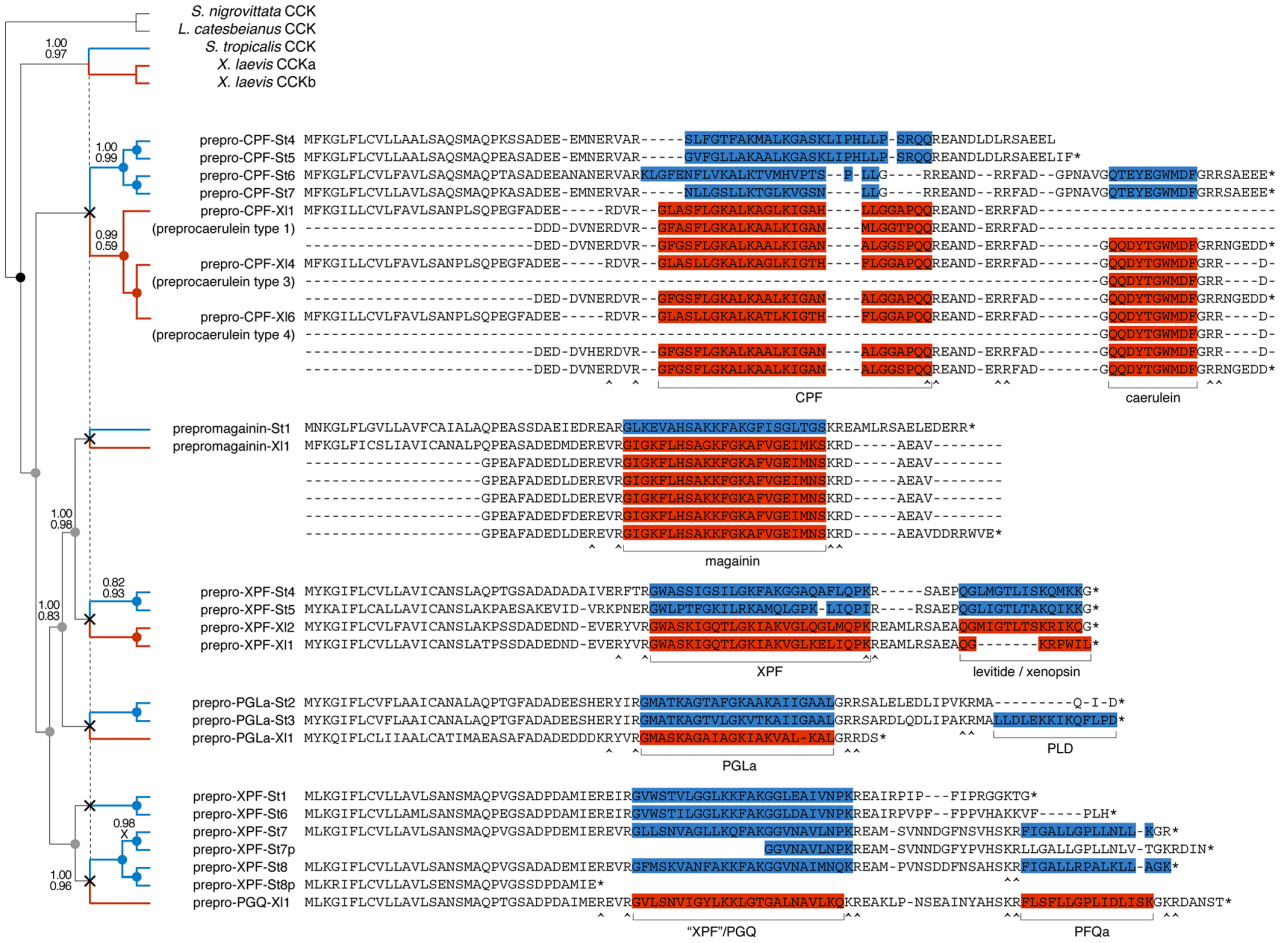
Evolutionary diversification of pipid AMP genes. Phylogenetic relationships of *S. tropicalis* and *X. laevis* AMP genes are shown in a consensus tree obtained by Bayesian analyses using the traditional two-step approach of phylogeny inference (separate alignment and Bayesian phylogeny inference) and using direct optimization (integrated Bayesian alignment and phylogeny inference). Each gene is represented here by its precursor protein sequence aligned to visualize similarity with its closely related homologues. Note that several of the *X. laevis* sequences occupy multiple lines because duplicated exons were aligned to each other. Unlabeled branches in the tree are supported by maximum posterior probabilities (1.00) under both methods; branches that received less support by one or both methods are labeled by their posterior probability under the two-step method (top) and under direct optimization (bottom). Nodes representing gene duplication events are labeled by circles and color-coded as follows: black, the split between *cck* and the ancestral pipid AMP gene; grey, gene duplication in an ancestor of *Silurana* and *Xenopus*; blue, gene duplication in *Silurana*; and red, gene duplication in *Xenopus*. Crosses linked by a vertical dashed line mark the divergence of *Silurana* and *Xenopus*, the resulting orthologous gene lineages are marked by blue and red branches respectively. Peptides in the precursor proteins are color-coded accordingly.

We reconstructed the evolution of the pipid AMP arsenal by phylogenetic analyses of the genes of *S. tropicalis* and *X. laevis* using amphibian *cck gene* sequences as outgroups. Bayesian analyses based on fixed alignments [49] or via direct optimization (integrating sequence alignment and tree estimation in a single analysis [50]) yield a well-resolved gene tree elucidating the rise of the AMP arsenal with respect to pipid evolution (Fig. 4). First, expansion of the gene cluster was well underway prior to the divergence between *Silurana* and *Xenopus*, which has been estimated by molecular clock analyses to have taken place approximately 42 (30–59) million years (Myr) ago [51]. At least five gene duplication events (grey nodes in Fig. 4) followed the basal gene duplication of the *cck* gene (black node) and predated the *Silurana*-*Xenopus* split, implying that the most recent ancestor of both genera had at least six AMP genes (X-marks in Fig. 4). Second, five well-supported branches in the tree identify the orthologous peptides in *S. tropicalis* of all known *X. laevis* AMPs and HLPs. For example, the two synergistic AMPs magainin-St1 and PGLa-St2 are confirmed to be orthologous to magainin-Xl2 and PGLa-Xl1, respectively, two *X. laevis* peptides with a similar synergistic effect [37], [38]. Levitide-St1 and levitide-St2 are confirmed to be orthologues of *X. laevis* levitide. Unexpectedly, the structurally distinct *X. laevis* xenopsin is recovered as closer related to *X. laevis* levitide than the *S. tropicalis* levitides, suggesting that this HLP descended from a levitide-like ancestral peptide after the *Xenopus*-*Silurana* split. Third, the tree indicates that several *Silurana* genes, after the divergence from *Xenopus*, underwent parallel gene duplication, yielding multiple pairs of closely related sister genes. Nine gene duplication events occurred in the *Silurana* lineage after it divergence from *Xenopus* (blue nodes in Fig. 4). In contrast, only three gene duplication events are observed for *Xenopus* since its divergence from *Silurana* (red nodes in Fig. 4).

#### Selection on the AMP precursor proteins

**Figure 5:**
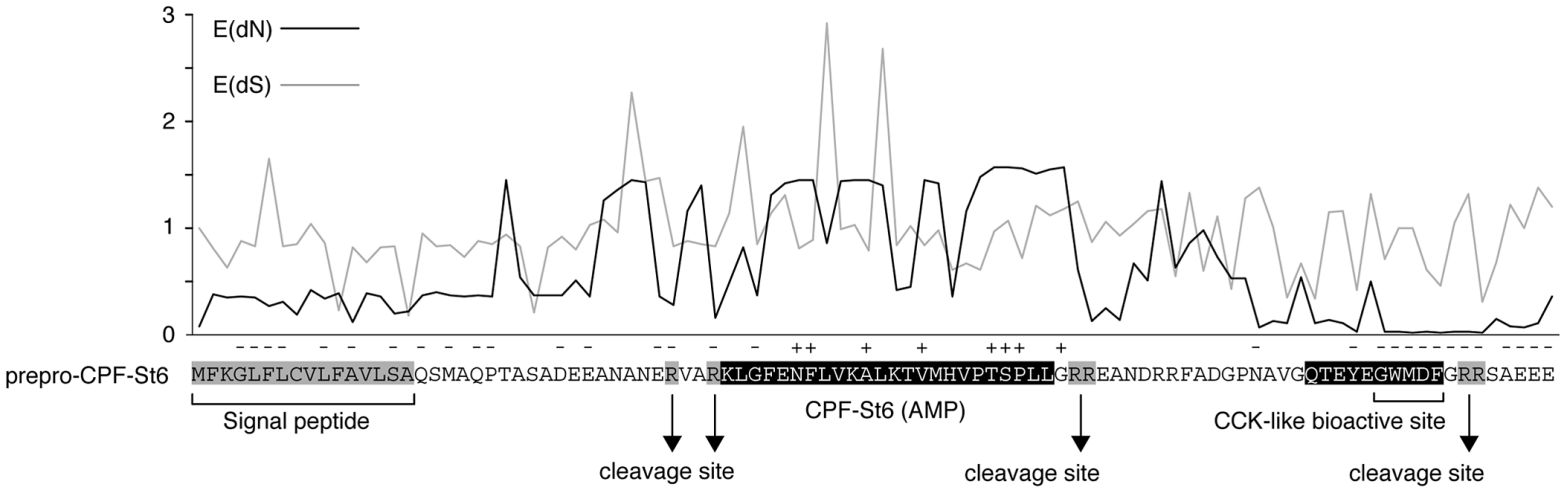
Patterns of diversifying and purifying selection in pipid AMP/HLP precursor proteins. Site-specific mean posteriors of nonsynonymous (E(dN), black line) and synonymous (E(dS), grey line) codon substitution rates are plotted along an exemplary precursor protein sequence (prepro-CPF-St6). Sites for which the black plot line rises above grey one have dN/dS ratios >1 (suggesting diversifying selection); those for which the black plot stays below the grey have dN/dS ratios <1 (suggesting purifying selection). Sites showing significant evidence of diversifying or purifying selection (at Bayes factor >50) are labeled with ‘+’ and ‘−’, respectively.

Alignment of the AMP/HLP precursor proteins indicates variation in amino acid substitution rates along their length (Fig. 4) in a pattern that is consistent with those observed in the AMP precursors of other amphibians [9], [10]. Codon-based likelihood analyses comparing sitewise nonsynonymous and synonymous substitution rates confirm that this rate variation reflects mixed patterns of diversifying (positive) and purifying (negative) selection (Fig. 5). High site-specific ratios of nonsynonymous over synonymous substitution rates are mostly observed for the spacer- and AMP-encoding regions, and all sites that are identified as showing significant evidence of diversifying selection lie in the these regions. This pattern may be consistent with selective pressures for a large structural diversity in amphibian AMP arsenals in adaptation to target a diverse spectrum of microorganisms [9], [10]. Conversely, sites with low site-specific ratios of nonsynonymous over synonymous substitution rates and significant evidence of purifying selection are concentrated in the regions encoding the signal peptide, peptide cleavage sites and the CCK-like bioactive site preserved in the *X. laevis* caerulein genes, *cpf-St6*, and *cpf-St7* (Fig. 5). Hence, similar to the evolutionarily conserved sequence motifs in the AMP gene promoter regions, parts of the coding sequences are subject to purifying selection, most likely to retain proper processing of the defense peptides.

## Discussion

By combining transcriptome and genome data with peptidome analyses, we present the first overview of the defensive peptide arsenal of an amphibian and the gene repertoire that underlies its synthesis. Together, the 13 charted genes encode up to 19 different peptides, a diversity comparable to, or even larger than those currently known from other pipid frogs, but far less extensive than the peptide arsenals reported from ranid frogs of the genus *Odorrana*, where transcripts of a single species encode for a diversity of 107 AMPs (55 AMPs in a single individual) [11], [52]. Our cDNA library further suggests variation in the level of transcription of individual genes, as indicated by respective numbers of randomly sequenced clones (Table 1). This pattern suggests that all AMP genes may not contribute equally to the arsenal of a single frog at any time, and may show both temporal and/or individual variation in expression.

Although our assays indicate that several of the *S. tropicalis* peptides are capable to kill a broad range of microorganisms at micromolar concentrations, the level of protection provided by the entire AMP arsenal against typical amphibian pathogens remains unknown. For example, unlike *X. laevis*, *S. tropicalis* has been suggested to be susceptible to the widespread chytrid fungus *Batrachochytrium dendrobatidis* [53], [54]. Microarray studies have shown that skin infection with this fungus hardly induces an immunogenetic response in *S. tropicalis*, and does not stimulate the expression of any AMP gene [55], [56]. This finding may indicate that the *S. tropicalis* AMP arsenal failed to protect against this fungus because its gene repertoire was not stimulated to an effective level in the first place.

Several authors have recently questioned the singular role of amphibian AMPs as first-line antimicrobials [12]–[16]. The weak activity of some AMPs compared to others has been interpreted as an indication that they evolved another, yet unknown function [17], [20]. On the one hand, there are indications that AMPs play a far more integrated role in the innate immune system, e.g. by acting as chemoattractants for immune cells or as modulators of cytokine release [11]. On the other hand, the cytolytic effect of AMPs on vertebrate cells, and their frequent co-expression with HLPs in various frog families may indicate that they serve a role in antipredator defense [35]. Our analyses confirm that they show activity against vertebrate red blood cells as well as T-cells at micromolar concentrations. At lower concentrations however, these AMPs may be ineffective in killing vertebrate cells, and instead, may serve a more subtle function. Some AMPs for example, are capable to stimulate insulin release by beta cells at nanomolar concentrations [57]. Although findings like these have a clear pharmaceutical potential, it remains uncertain whether such activity provides an adaptive benefit to amphibians when being attacked by a predator. Regardless of other potential adaptive functions, it seems unlikely that the broad-scale antimicrobial activity of some AMPs would not provide any adaptive benefit to the host species, even if it fails to provide protection to some pathogens.

### 1. Differential evolution of the AMP/HLP arsenal in *Silurana* and *Xenopus*

The phylogeny of the pipid AMP/HLP arsenal is consistent with a birth-and-death model of gene evolution, similar to those implicated for vertebrate immune gene families [58] and reptile toxin genes [59], [60], in which some gene lineages survive for a prolonged time, and may give rise to additional genes through subsequent duplication, while others are eventually lost. Gene loss is confirmed by the presence of several incomplete genes in the *S. tropicalis* cluster, one of which shows clear signs of pseudogenisation (*cpf-St8b*), and the apparent absence of orthologues of *xpf-St1* and *xpf-St6* in *X. laevis*. Comparative peptidome analyses of several *Silurana* and *Xenopus* species have recently indicated that ancient genome duplication events did not result in an increased AMP diversity in allopolyploid species [16], [17], [20]. This observation led to the conclusion that polyploidization events were compensated by subsequent gene losses, counterbalancing the expected doubling of the AMP arsenal. Our analyses confirm similar levels of AMP diversity in *S. tropicalis* and *X. laevis* but also reveal that the comparable arsenals in both taxa arose under contrasting patterns of gene evolution. The diploid *S. tropicalis* has the largest AMP/HLP gene repertoire (due to higher rates of tandem gene duplication or lower rates of gene loss) but each gene encodes a single AMP and/or a single HLP. Instead, *X. laevis*, despite being tetraploid, has fewer genes (due to lower rates of gene duplication or increased gene loss), but several of them (e.g. the *magainin* and *caerulein* genes) are characterized by multiple tandem-repeated peptide–encoding exons. The difference between both patterns may have been maintained by the self-sustaining nature of tandem repeats (whether genes or exons), because as their number increases, so does the probability of unequal crossing-over among neighboring copies, potentially creating additional duplicates of the same type. Exon duplication is considered an important mechanism of transcriptional economy by which peptide diversity can be increased, either through alternatives splicing [61] or by differential cleavage of tandem-encoded peptides [62]. However, duplicated exons in the *caerulein* and *magainin* genes show quasi-zero sequence divergence (Fig. 4), possibly reflecting very recent duplication events, extremely low evolutionary rates, or gene conversion. Alternative splicing therefore adds little to peptide diversity, but in the *X. laevis* genes encoding multiple tandem-repeated exons may provide a way to boost the synthesis of peptides at a low transcriptional cost.

### 2. Dynamic structural and functional evolution of the pipid AMP gene repertoire

**Figure 6:**
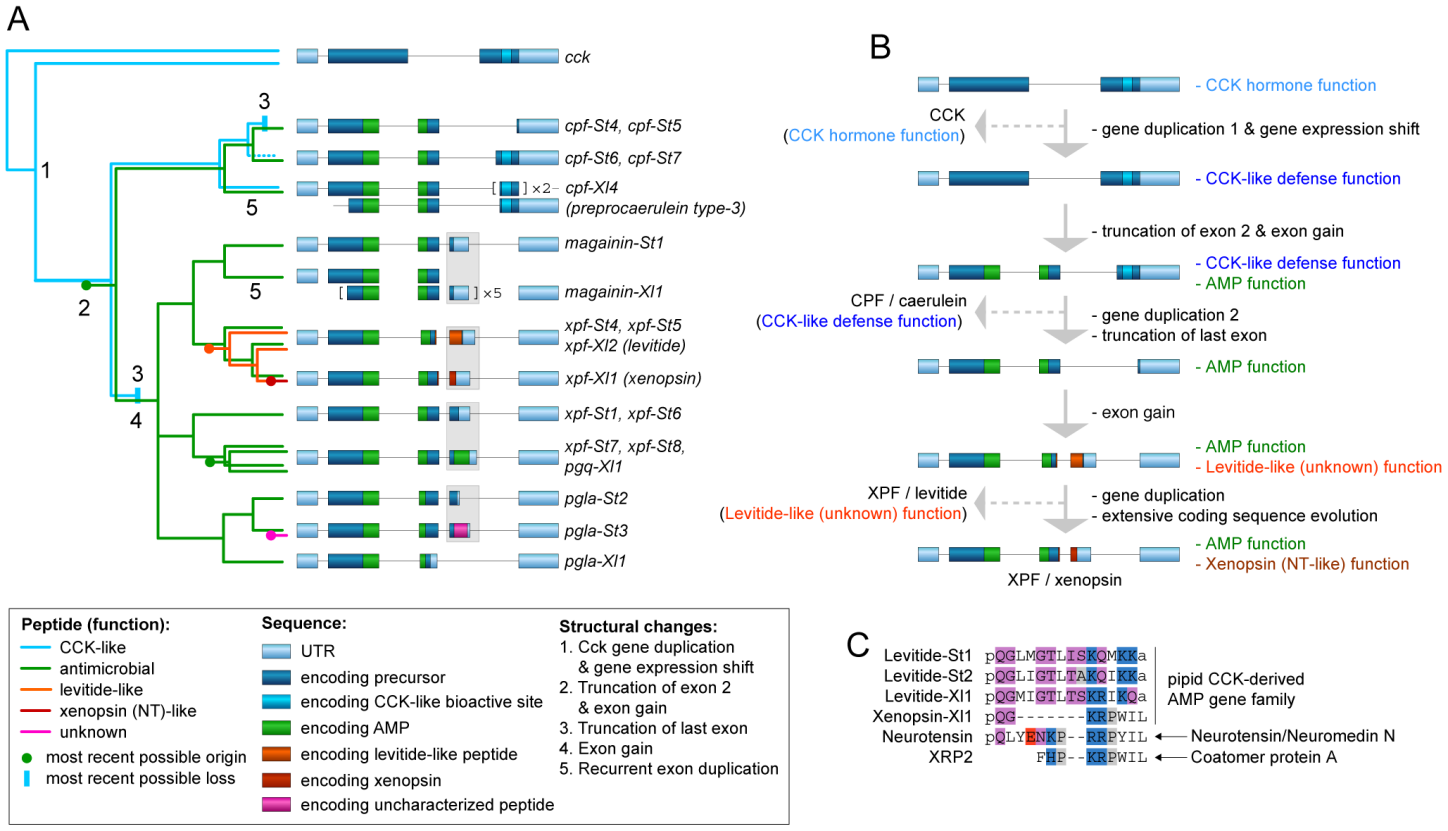
Structural and functional evolution of the pipid defense peptide arsenal. (A) Evolutionary scenario for the origin and loss of defense peptides and their function along a summarized phylogenetic tree. Numbers along tree branches represent structural changes as mentioned in the legend. (B) Evolutionary trajectory showing structural changes and functional transitions from the ancestral *cck* gene to the present-day *X. laevis xpf-Xl1* gene, encoding the AMP XPF and the HLP xenopsin. Only gene duplication events relevant to functional changes (see text) are indicated. (C) Comparative alignment of levitide-like peptides, *X. laevis* xenopsin and the vertebrate neurohormones with which it shows evolutionary convergence. The origins of the different peptides are indicated on the right. Amino acids are color-coded as follows: white, hydrophobic; grey, near-neutral; purple, uncharged polar; blue, cationic (basic); red; anionic (acidic).

Our phylogenetic analyses allow us to formulate an evolutionary scenario for the timing of origin and loss of specific peptide types and their corresponding functions in light of major structural changes in the pipid AMP gene repertoire (Fig. 6). The first major step in the evolution of the peptide arsenal entails the transition of a neurohormone (CCK) to a basal defense peptide (Figs. 6A, 6B). The HLP caerulein, encoded by three genes in *X. laevis*, contains the same bioactive site as CCK and shares its capacity to bind vertebrate CCK receptors, thereby inducing pancreatitis, vomiting, diarrhea, hypotension, and inhibition of exploratory and feeding behavior [63]. In addition, C-terminal sequences homologous to caerulein are retained in the *S. tropicalis* precursors prepro-CPF-St6 and prepro-CPF-St7. Together, these *Xenopus* and *Silurana* genes represent an early-diverged lineage in the pipid AMP gene tree. It is therefore likely that caerulein retained the basal defense function of the *cck*-derived gene family. Its origin was accompanied by a gene duplication event (gene duplication 1 in Fig. 6B) and a shift in expression from the gastrointestinal tract and brain to the granular skin glands. The taxonomic distribution of newly identified promoter elements (Fig. 3) provide an indication that at least part of the changes required for this expression shift may have happened long before the *cck* gene duplication, implying that the ancestral *cck* gene was “preconditioned” to acquire a skin-secretory function.

A second major step involves the origin of a cationic alpha-helical AMP in the central region of the ancestral precursor protein, leading to a bifunctional AMP/HLP gene product with an architecture similar to, e.g. the present day prepro-CPF-St6 (Figs. 6A, 6B). This step is marked by truncation of the N-terminal spacer region, and the origin of an additional exon, encoding the C-terminal half of pipid AMPs. In contrast, an evolutionary conserved -RXXR- site, inducing the N-terminal cleavage of the longest functional CCK-isoforms in vertebrates (e.g. CCK-58 in humans) has been preserved in the majority of pipid AMP precursors, where it induces cleavage of the functional AMPs.

Third, after establishment of an antimicrobial function, the basal CCK-like defense function was lost twice independently (Fig. 6A), yielding monofunctional AMP precursors with an architecture similar to e.g., the present-day prepro-CPF-St5 precursor (Figs. 6A, 6B). One loss occurred in the gene ancestral to *cpf-St4* and *cpf-St5*, while a second loss happened in the gene ancestral to *magainin-St1* through *pgla-St3*. In both cases, the loss of the CCK-like function followed gene duplication events and were accompanied by the 5′-truncation of the genes' last exon, eliminating the sequence encoding the CCK-like bioactive site.

Fourth, after loss of the basal CCK-like defense function, new AMPs and HLP functions (pFQa-like peptides, levitide-like peptides, PLD-St1), arose alongside the antimicrobial function, again yielding a bifunctional gene. All of these secondary peptides are encoded by an extra exon (exon 4 in *xpf-St4*, *-St5*, *-St-6*, *-St7*, and *-St8*, and *pgla-St2*, and *-St3*), but due to their lack of structural similarity, it is unclear whether they originated independently, or share a common origin. The latter would imply extraordinary structural diversification of the peptides due to extremely high substitution rates in exon 4.

On at least two occasions, evolution of the pipid AMP genes gave rise to striking cases of evolutionary convergence in peptide sequence. One case is represented by caerulein, which independently evolved to an identical structure in *Litoria* frogs, starting from a different neurohormone (gastrin) [30]. A second example is represented by xenopsin, which according to our phylogenetic analyses evolved from a levitide-like peptide (Figs. 4, 6A, 6B). Apart from AA substitutions, this evolutionary process involved a 7-AA deletion in the peptide and loss of C-terminal amidation (Fig. 6C). Xenopsin shows strong structural and functional similarities to the hormones neurotensin (NT) and xenopsin-related peptide 2 (XRP-2), two universal NT receptor agonists in vertebrates [26], [64], [65]. These hormones share five and six C-terminal AAs with xenopsin, respectively, all of which are involved in neurotensin receptor binding [65]. Effects of NT and XRP-2 include stimulation of exocrine pancreatic secretion, suppression of food intake, increase of vascular permeability (enhancing inflammation responses and hypotension), and stimulation of histamin release by mast cells (enhancing an allergenic reaction) [66], [67]. While NT is cleaved from its own precursor along with the hormone neuromedin N [68], XRP-2 is cleaved from the N-terminal region of Coatomer Subunit alpha (CopA), a 138-kDa intracellular protein [69]. Because xenopsin is unrelated to either, its evolution is likely to reflect adaptive convergence to neurotensin receptor binding.

### 3. The pipid AMP arsenal as a model system of functional innovation

Toxin gene families showing complex functional diversity among their members in venomous animal taxa have been used as model systems to investigate the role of gene duplication and diversifying selection in functional innovation, and lended support to different theoretical models of neofunctionalisation [59], [70], [71], [72]. The pipid AMP/HLP arsenal provides an opportunity to extend the same theoretical framework to amphibian defense peptide arsenals and presents an empiricial illustration of how peptide-encoding genes may acquire new functions independent of gene duplication.

Current theoretical models invoke gene duplication as a key factor of functional innovation. Under the original model of neofunctionalisation (“mutation during nonfunctionality”, [73]) gene duplication delivers a functionally redundant gene, which, released of selective constraints to maintain the ancestral function, accumulates mutations that lead to a new function. Alternative models instead invoke subfunctionalisation following gene duplication to divide multiple functions of the parental gene over the duplicates in a nonadaptive way (“duplication-degeneration-complementation” ; [74]), or to resolve adaptive conflicts between different ancestral functions (“escape from adaptive conflict” [75], [76]). Although gene duplication of the *cck* gene allowed the origin of a CCK-like defense function in a pipid ancestor, it did not trigger subsequent functional innovation. Instead, new functions recurrently arose by mutation of a precursor protein, resulting in the posttranslational processing of a secondary peptide (an AMP) alongside the original one (e.g. a CCK-like HLP). The resulting bifunctional gene is consistent with the starting condition in several theoretical models invoking subsequent gene duplication [74], [76], [77]. However, pipid AMP/HLP genes deviate from these models (or represent a special case) by attaining multifunctionality through the production of multiple peptides, rather than a single peptide or protein with multiple functions.

One implication of this mechanism is that genes in the AMP/HLP arsenal acquired new functions while being under purifying selection to retain an older one. This could have been possible by mutations in a gene region that is subject to only marginal purifying selection [10]. Selection on a gene to retain its original function is likely to affect only regions of the gene that are crucial for the proper expression, processing and functioning of the peptide [10], [78]. In the case of the Pipid AMP/HLP gene repertoire, this is confirmed by purifying selection on gene regulatory elements, and exon regions encoding signal peptide, cleavage sites and the CCK-like bioactive site (Figs. 3 and 5). The evolution of a new functional peptide in precursor regions under relaxed selection thereby not avoids adaptive conflicts with the original peptide but may even rely on the gene regions under purifying selection to obtain an adaptive value.

Another implication is that selection on a gene to retain its original function is likely to affect the probability of possible new functions to arise. In pipid frogs, purifying selection to retain a gene with all the necessary features to produce a skin-secretory peptide, increases the probability that any new function will be related to skin secretion as well. Therefore, while representing a red line through the evolutionary history of the pipid AMP gene repertoire, skin gland expression acted as a key determinant of functional innovation.

Although gene duplication did not act a the direct trigger of functional innovation, it may have subsequently still led to subfunctionalisation [74], [75], [76], by relaxing selective pressures to retain both functions in each daughter gene. Subfunctionalisation may explain the convergent loss of the original CCK-like defense function in multiple gene lineages after duplication, completing the final step in their functional transformation from HLP gene to AMP gene (Fig. 6B).

The pipid defense peptide arsenal illustrates an evolutionary mechansim independent of gene duplication that could explain the presence of precursor proteins yielding multiple peptides with distinct structures and bioactivities in the poison or venom glands of various animals. Transcripts of a single gene isolated from *Bombina* toad skins encodes both bradykinin peptides and a structurally unrelated bradykinin inhibitor [79], while natriuretic peptide precursors produced in the venom glands of vipers and *Heloderma* lizards are further cleaved to obtain angiotensin-converting enzyme (ACE) inhibitors, and bradykinin receptor antagonists, respectively [80], [81]. Furthermore, the evolution of multiple peptides within a single precursor, when followed by gene duplication and subfunctionalisation, provides an explanation for the existence of closely related genes encoding structurally and functionally distinct peptides. Homologous genes encoding fundamentally different peptides, like AMPs, bradykinin-like HLPs and opioid peptides, have been identified in ranid and hylid frogs [3], [10], [82], [83]. A recent evolutionary study of snake venom metalloproteinases (SVMPs) showed how differential protein domain loss following gene duplication gave rise to proteins with distinct toxin functions [70]. This study implied that the ancestral SVMP toxin was composed of multiple domains, each with a distinct function, but did not explain how these domains were combined in a single protein.

## Materials and Methods

### 1. Ethics statement

The experiments, maintenance and care of mice complied with the guidelines of the European Convention for the Protection of Vertebrate Animals used for Experimental and other Scientific Purposes (CETS n° 123). The experiments for this study were approved by the Ethical Committee for Animal Experiments of the Vrije Universiteit Brussel, VUB, Brussels, Belgium (Permit Number: 08-220-8).

### 2. Charting the *S. tropicalis* AMP gene repertoire

Freshly dissected skin tissue of *S. tropicalis* was snap-frozen in liquid nitrogen, ground to powder with mortar and pestle, and stored at −80°C. mRNA was extracted using the Qiagen Oligotex mRNA midi kit and cDNA libraries were made using the Clontech Creator SMART cDNA library construction kit. Plasmids with cDNA inserts were cloned in Oneshot Electrocompetent GeneHog *E. coli* cells (Invitrogen Corp.) and 384 randomly selected clones were sequenced by the Australian Genome Research Facility. Clones corresponding to AMP-encoding transcripts were identified by BLAST searches based on sequence homology to mRNA and gene sequences encoding known peptides in *X. laevis*. Subsequent online translation of these transcripts yielded the corresponding precursor sequences. Functional peptides in the precursors were predicted by comparison with previously described peptides from various *Silurana* and *Xenopus* species.

The *S. tropicalis* AMP gene repertoire was charted by BLAST-searches against the *Xenopus tropicalis* 4.1 genome (DOE Joint Genome Institute; http://genome.jgi-psf.org/Xentr4/Xentr4.home.html; *X. tropicalis* is the previous name of *S. tropicalis*) using the newly obtained transcripts as queries. Based on a large sequence overlap including *pgla-St2* and *pgla-St3*, we concatenated genomic scaffolds 665 and 811. We assume that these scaffolds were kept separate during contig assembly due to sequence gaps whose lengths were improperly estimated, resulting in apparent sequence conflicts. Intron/exon boundaries of identified AMP genes were checked by verifying the presence of GT/AG intron borders flanking the inferred exons.

### 3. Tracing predicted AMPs and HLPs in the *S. tropicalis* skin peptidome

Freshly dissected dorsal skin tissue of *S. tropicalis* frogs were placed in a 90∶9∶1 (v∶v∶v) CH_3_OH/H_2_0/HCOOH solution and stored at −20°C. After sonication (5 times 2 min) and centrifugation (10 min at 9000 *g*), the supernatant was lyophilized (SpeedVac Concentrator). Dry peptide extracts were reconstituted in 100 µL of 0.1% aqueous HCOOH and lipids were removed by re-extraction with equal volumes of ethyl acetate and n-hexane respectively. The sample was filtered through a 0.22 µm spin-down filter (Ultrafree-MC; Millipore) and 1/10^th^ of the sample was analyzed by nanoLC-MS/MS on an Ultimate 3000 Nano and Capillary LC System (Dionex) coupled to a microTOF-Q mass spectrometer (Bruker Daltonics GmbH). The peptides were separated on a Pepmap C18 column (3 µm, 75 µm×150 mm, LC Packings) using a 50 min linear gradient from 95% solvent A and 5% solvent B to 50% solvent A and 50% solvent B at a flow rate of 200 nl/min (solvent A: deionised water containing 0.1% HCOOH; and solvent B: CH_3_CN containing 0.1% HCOOH). In the mass spectrometer, doubly or triply charged ions of sufficient abundance are selected for fragmentation by the software (MS/MS). [84]. MS/MS peak list files were submitted to an in-house version of MASCOT server (Matrix Science, USA) and screened against a database of predicted AMP/HLP precursor protein sequences. This way, the actual cleavage and posttranslational processing of predicted peptides from the precursors was verified. The resulting spectra and corresponding tables are provided in Text S2.

### 4. Structural and functional analyses of *S. tropicalis* AMPs

Secondary structure of AMPs was predicted using a neural network algorithm as implemented on the Psipred server [32]. Peptides predicted to have an alpha-helical structure were projected in a wheel projection to visualize their amphipathic nature. Hydrophobic moments were calculated using the combined consensus scale implemented in HydroMCalc [85].

Seventeen predicted and recovered peptides were *de novo* synthesized using solid-phase technology by CASLO Laboratory ApS (Lyngby, Denmark) and delivered as HPLC-purified (>95%) trifluoracetate (TFA) salts. The peptide salts were stored as 5.12 mM stock solutions in 0.01% (V/V) acetic acid/0.2% (m/V) BSA. Peptides were tested against *Escherichia coli* (ATCC 25922) and *Pseudomonas aeruginosa* (ATCC 15692), *Staphylococcus aureus* (ATCC 25923), *Micrococcus luteus*, *Saccharomyces cerevisiae*, a protozoan parasite (*Trypanosoma brucei brucei*), mouse (*Mus musculus*) red blood cells and mouse spleen tissue cultures (containing 50–60% B-cells, ∼25–30% T-cells, and 5% myeloid cells). All assays were performed in duplicate or triplicate.

Activity against the bacteria and fungus was measured by assessing the lowest peptide concentration in a series of twofold dilutions at which no growth was detected (known as the minimum inhibitory concentration, MIC). Bacterial and yeast cultures (5×10^5^ colony forming units/ml) were prepared in Müller-Hinton (MH) broth and Luria-Bertani (LB) broth respectively and transferred to serial dilutions of peptides ranging from 512 or 256 µM down to 1 or 0.5 µM in 96-well polypropylene plates. After incubation at 37°C (bacteria) or 30°C (*S. cerevisiae*) for 18 hours, growth of the cultures was checked by eye. In addition to MIC, we determined minimum microbicidal concentrations (MMC) by inoculating samples of the MIC assays on agar plates lacking peptides and checking for overnight growth. In all cases, MIC and MMC were identical.

Activity against *T. brucei* parasites was assessed as the minimum concentration in a series of twofold dilutions required to kill 95% of parasites in 30 minutes (LC_95_). Frozen stabilates of *Trypanosoma brucei brucei* AnTat1.1 bloodstream parasites were expanded by infection of C57Black/6 mice (Janvier). Mice with systemic parasitaemia (typically 4–5 days post infection) were exsanguinated and parasites were purified from heparinized blood by DEAE-cellulose (DE52, Whatman) chromatography. Collected parasites were washed twice with Phosphate-Saline-Glucose buffer (PSG-buffer) and enumerated microscopically using a Bürker hematocytometer. The DEAE52-purified parasites (stock: 10^6^ parasites/ml PSG buffer containing 5% FCS) were added to polypropylene 96-well plates containing the serial peptide dilutions to obtain total volumes of 200 µl. After incubation for 30 minutes, trypanolytic activity was assessed using a light microscope by calculating the percentage of dead parasites in a minimum of 200 counted cells.

Activity against mouse red blood cells was examined by assessing the lowest peptide concentration in a series of twofold dilutions causing at least 50% hemolysis (HC_50_). Briefly, heparinized blood was obtained by cardiac puncture of mice euthanized via CO_2_. The blood was diluted 1/100 in RPMI and added to polypropylene 96-well plates containing the serial peptide dilutions to obtain total volumes of 100 µl. The plates were incubated at 37°C in a humidified atmosphere for 30 minutes and centrifuged at 1400 rpm for 2 minutes to pellet intact red blood cells allowing visual observation of hemolysis. Supernatants were subsequently transferred to 96-well flat-bottom plates to allow spectrophotometry using an ELISA reader (OD measured at 550 nm). The percentage of hemolyis for each peptide concentration was calculated as 100×(OD_obs_−OD_0%_)/(OD_100%_−OD_0%_), where OD_obs_ is the OD measured for the peptide concentration, OD_0%_ is the average OD in the absence of peptides (0% hemolysis), and OD_100%_ is the average OD in the presence of 1% Tween-20 (100% hemolysis).

Activity against T-lymphocytes was assessed as the lowest peptide concentration in a series of twofold dilutions causing at least 50% inhibition of Concanavalin A-induced T-cell proliferation (IC_50_). Solutions containin 2×10^5^ naive C57Black/6 splenocytes in the presence of 2.5 µg Concanavalin A were added to polypropylene 96-well plates containing the serial peptide dilutions to obtain total volumes of 100 µl. After incubation for 24 hours at 37°C in a humidified atmosphere, the cells were pulsed with [^3^H]thymidine and incubated for an additional 18 hours. The amount of [^3^H]thymidine incorporation was assessed by a β-counter as a measure of cell proliferation and the percentage of proliferation inhibition for each peptide concentration was calculated as 100×(1−(T_obs_/T_100%_)), where T_obs_ is the [^3^H]thymidine level measured for the peptide concentration, and T_100%_ is the [^3^H]thymidine level measured for Concanavalin-A-induced cell proliferation in the absence of peptide (representing 100% proliferation).

### 5. Analysis of gene promoter regions

Intergenic regions in the cluster were screened for the presence of evolutionary conserved elements (phylogenetic footprints) using the program Tracker [86]. Tracker identified the gene promoter regions as only conserved non-repeat regions in between adjacent AMP genes. The 500-bp upstream regions of all *S. tropicalis* AMP genes, four *X. laevis* AMP genes and the *cck* genes of *five* vertebrates were aligned using the EINSI algorithm in MAFFT 6.704 [87]. Candidate regulatory elements in the gene promoter regions were identified by: (1) determining aligned sites with more than 75% sequence conservation across all AMP genes, and (2) by searching for significantly overrepresented sequence motifs in the promoter regions using MEME 4.8.0 [42].

### 6. Phylogenetic reconstruction of pipid AMP gene diversification

AMP gene sequences retrieved from the *S. tropicalis* genome were combined in a single dataset with related gene and mRNA sequences of *X. laevis* retrieved from GenBank. Five amphibian *cck* gene or mRNA sequences were added to serve as outgroups. Phylogenetic relationships were estimated using the conventional ‘two-step’ approach, involving sequence alignment and tree reconstruction as separate steps of phylogeny inference, and a ‘direct optimization’ approach that accounts for alignment uncertainty by integrating sequence alignment and tree construction in a single algorithm. For the two-step approach, sequences were aligned using MAFFT. To avoid the erroneous alignment of nonhomologous sequences, exons were aligned separately and subsequently concatenated. Phylogenetic analyses were performed with MrBayes 3.1.2 [49] using a GTR+G+I model of DNA substitution. Two parallel runs of four incrementally heated (temperature parameter = 0.2) Markov chain Monte Carlo (MCMC) chains were performed, with a length of 10,000,000 generations, a sampling frequency of 1 per 1,000 generations, and a burn-in corresponding to the first 2,000,000 generations. Convergence of the parallel runs was confirmed by split frequency standard deviations (<0.01) and potential scale reduction factors (approximating 1.0) for all model parameters, as reported by MrBayes. Adequate posterior sampling was verified using Tracer 1.5 [88], by checking if the runs had reached effective sampling sizes >200 for all model parameters.

Bayesian analyses under direct optimization were conducted with BAli-Phy 2.0.2 [50]. Alignment constraints were imposed to maintain gene alignments that respect exon boundaries. The data set was analyzed under a GTR+G+I model of DNA substitution combined with a RS07 model of DNA insertion/deletion. Four independent analyses of a single MCMC chain each were run for ten million generations, and alignments and trees were sampled every 1000 generations. Again, convergence of the runs, and effective sampling size of the log-likelihood values and model parameters was checked using Tracer.

### 7. Selection analyses

We investigated whether diversifying (positive) or purifying (negative) selection affected the evolution of coding sequences in the expanding gene cluster using the random effects likelihood (REL) method, as implemented in the HYPHY software package [89] Kosakovsky Pond et al., 2005]. This method is considered the most suitable for datasets <50 sequences, draws sitewise synonymous and nonsynonymous codon substitution rates from separate rate heterogeneity distributions, and implements an empirical Bayes approach to test for significant selection at any specific site [90]. As such, REL represents an extension of the methods implementend in the benchmark program PAML [91]. Analyses were conducted using a MG94 codon substitution model ‘crossed’ with a GTR nucleotide substitution model, and codon sites were identified as subject to significant diversifying or purifying selection at Bayes factors >50. Due to the gain, loss and truncation of exons in the history of pipid AMP genes, the selection analyses were conducted on separate data sets for exon 2 (combining CCK and AMP gene sequences), exon 3 (composed of AMP gene sequences only) and the last exon (composed of CCK gene sequences and the homologous sequences of four AMPs that encode a CCK-like bioactive site).

## Supporting Information

Text S1 FASTA file containing the mRNA sequences of the 13 transcriptionally active *Silurana tropicalis* AMP genes. Coding regions are written in blue; untranslated regions are written in red.
(DOC)

Text S2 Results of an MS/MS ion search against a database of *Silurana tropicalis* AMP/HLP precursor proteins predicted from cDNA and gene sequences, using an in-house version of MASCOT Server (Matrix Science, USA). Individual ions scores >26 indicate identity or extensive homology (p<0.05). For each of the confirmed peptides, the MS/MS fragmentation spectrum and corresponding table with fragmentation masses is shown. a, C-terminal amidation; Mr, molecular weight; pQ, pyroglutamate.
(DOC)
