# Supplementary material for: Origin and Functional Diversification of an Amphibian Defense Peptide Arsenal
Source: PLoS Genet. 2013 Aug 1;9(8):e1003662. doi: 10.1371/journal.pgen.1003662 (PMC3731216; doi:10.1371/journal.pgen.1003662)
Supplement: Text S1 — FASTA file containing the mRNA sequences of the 13 transcriptionally active Silurana tropicalis AMP genes. Coding regions are written in blue; untranslated regions are written in red. (DOC) [file pgen.1003662.s001.doc]

Supporting information: Text S1

FASTA file containing the mRNA sequences of the 13 transcriptionally active *Silurana tropicalis* AMP genes. Coding regions are written out in blue; untranslated regions are written out in red.

>Silurana tropicalis cpf-St4

tgaaaagcaactaactaactaactaagtgttttctttcatataattctgtcacctactgcaggttctctacagtgtttgaagtggactggaaaatctgaaacgaactggacttttttaatataattttaggtcattaggtcattaggtaacttttaaatacagcatgttcaaagggctatttctttgtgtactgttggctgcgctctcagcacagtcaatggcacagccaaaatcctctgcagatgaagaggaaatgaatgaacgagtagctcgaagtttgtttggaacatttgccaagatggctttaaaaggagcttcaaaattaattccacatttacttccatcaagacaacaacgggaagccaatgatctagatcttcgctctgctgaagaacttatcttttgaAATGCGATTGCACAATTCGTACAAGACTGCATTAAGGTTTGACAGTAGGAAGATTCATTTGAAAGCATCCAGACCTACAAAACTACCAGCCTGTTTCTGCAAATGATAAATAAACATAACTGAGCAAATTGAA

>Silurana tropicalis cpf-St5

ACAGCAATCTGTTCTACGCACCTTGCCAAAGCTCTCTTGAAAAGCAACTAACTTTTGAAAGCATGTTCAAAGGGCTATTTCTTTGTGTACTGTTGGCTGCGCTCTCAGCACAGTCAATGGCACAGCCAGAAGCCTCTGCAGATGAAGAGGAAATGAATGAACGAGTAGCTCGAGGTGTTTTCGGATTACTTGCCAAGGCTGCTTTAAAAGGAGCTTCAAAATTAATTCCACATTTACTTCCATCAAGACAACAACGGGAAGCCAATGATCTAGATCTTCGCTCTGCTGAAGAACTTATCTTTTGAAATGCGATTGCACAATTCGTACAAGACTGCATTAAGGTTTGACAGTAGGAAGATTCATTTGAAAGCATCCAGACCTACAAAACTACCAGCCTGTTTCTGCAAATGATAAATAAACATAACTGAGCAAATTGAA

>Silurana tropicalis cpf-St6

ATCACAGCTCTCCTTACTGAGCACCTTGCAAAATCTCTTGCAATTATCTTTGGAAAGCATGTTCAAAGGGCTATTTCTTTGTGTATTGTTTGCTGTGCTCTCAGCACAGTCAATGGCTCAGCCAACAGCCTCTGCAGATGAAGAGGCAAATGCAAATGAACGAGTAGCTCGAAAATTGGGATTTGAAAATTTTCTGGTAAAGGCTTTAAAGACTGTAATGCATGTGCCTACAAGTCCTTTATTAGGGCGACGGGAAGCCAATGATCGCCGCTTTGCTGATGGACCTAATGCAGTGGGTCAAACAGAGTACGAGGGTTGGATGGATTTTGGCCGCCGCAGTGCCGAAGAAGAGTAATACTGTTCTGGAGAACATTAAATATATAAAAATGCATCTGTTTCTGTACAAATGAAATAAAGCATTCCCTACC

>Silurana tropicalis cpf-St7

ATCACAACACTCCTTACTGAGCACCTTGCAAAATCTCTTGCAATTATCTTTGGAAAGCATGTTCAAAGGGCTATTTCTTTGTGTACTGTTGGCTGTGCTCTCAGCACAGTCAATGGCTCAGCCAAAAGCCTCTGCAGATGAAGAGGAAATGAATGAACGAGTAGCCCGAAATTTGCTGGGATCACTTCTGAAGACTGGTTTGAAAGTGGGTTCAAATCTTTTAGGAAGGCGAGAAGCCAATGATCGCCGCTTTGCTGATGGACCTAATGCAGTGGGTCAAACAGAGTACGAGGGTTGGATGGATTTTGGCCGCCGCAGTGCCGAAGAAGAGTAATACTGTTCTGGAGAACATTAAATATATAAAAATGCATCTGTTTCTGTACAAATGAAATAAAGCATTCCCTACCTGAAGGAATCT

> Silurana tropicalis magainin-St1

GCTATTGAAAGACAACTACATTTGGGAAGAATGAACAAAGGATTATTCCTCGGTGTATTACTTGCCGTGTTCTGTGCAATTGCACTGGCACAACCAGAGGCCTCTTCGGATGCGGAAATAGAGGACAGAGAGGCCCGGGGATTGAAAGAGGTAGCGCATTCTGCAAAAAAATTTGCAAAAGGTTTTATTTCAGGGCTTACAGGTTCAAAACGAGAGGCAATGCTTCGCAGTGCTGAGCTAGAAGATGAGAGGAGATGAGTTTCATGAAGAGGAAAGCAGCACTGAAGAACAACTATTTGTCTGAATATTTCTATGGACTGAATTTATTCTGAACAATGCGGTGCTGAAGAATAAGAAAATCCTGCAGATCCCCTAATACATATAGTATAACCACCTGCTCCTGTTAAGGGCAAATAAAATGAGCA

>Silurana tropicalis xpf-St4

ACAATCATCTGTATTTAGTACCTTACTACTTCTGTTACTGAGAAACACTACATTTTGAAAGGATGTACAAAGGGATATTCCTCTGTGTATTACTTGCTGTGATCTGTGCAAACTCCCTGGCACAGCCCACAGGCTCTGCAGATGCAGATGCAGATGCTATAGTCGAGCGCTTTACCAGAGGCTGGGCGAGTAGCATTGGCTCAATTTTGGGAAAATTTGCTAAAGGTGGAGCACAAGCATTCCTACAACCTAAACGCAGTGCTGAGCCCCAAGGTTTGATGGGTACACTGATAAGCAAGCAAATGAAAAAAGGATAAACAATAAAACTGCAGAAGAAGAAAAAGCATTTTACTGGAAATAATGTTGCAGGAAGAATACCGTTGTCCCTCAGATACCTGCCAGATCTCTATAAATACACCATAAACCAGCAAATAAAATGAGCAAAAATTAAGCA

>Silurana tropicalis xpf-St5

AGTGCAGTCATCTATATTTAGAACCTTGCTACTTCTGTTACTGAAAAACACTACATTTGGAAAGGATGTACAAAGCAATATTTCTCTGTGCATTACTTGCTGTGATCTGTGCAAACTCCTTGGCAAAGCCAGCAGAATCTGCAAAAGAAGTAATTGATGTCCGGAAGCCCAATGAGCGAGGCTGGTTACCTACATTTGGAAAGATTTTAAGAAAAGCTATGCAACTTGGACCAAAATTAATACAACCTATACGACGCAGCGCTGAGCCCCAAGGTTTGATAGGTACACTGACAGCCAAGCAAATTAAAAAAGGATAAAAAGAAGAGCTACGCTGGAGAAAAAGCATTTTCATGGAAATAATGCTGCAGGGCTGAAGAATAAAGTTGTCCCCAGATACCTGCCAGATCTCTACAAATACACCATTAAAGAGCAACTAAAATGAGCAAAATATATGAAATAAAATAAGCAAAACCTCT

>Silurana tropicalis xpf-St1

AAGCATTTATACTGAGCACCTTGCCGTTTGTGTGACAGAAAAGGATCTACATTTGGAAAGAATGTTGAAAGGAATATTCCTCTGTGTATTACTTGCTGTGCTCTCTGCAAACTCAATGGCACAGCCAGTAGGCTCTGCAGACCCAGATGCGATGATTGAACGAGAAATTCGGGGAGTATGGAGTACTGTTCTGGGTGGTTTAAAAAAATTTGCAAAAGGTGGTCTTGAGGCTATAGTGAATCCAAAACGAGAAGCAATAAGGCCCATCCCTTTTATACCTCGCATCTCCCTAAACCTATAAATATAAATAACAAATGCTGGAAAA

>Silurana tropicalis xpf-St6

CAAAGCATTTATACTGAGGACCTTGTCGTTTGTGTGACAGAAAAGCAGCTACATTTGGAAAGAATGTTGAAAGGAATATTCCTCTGTGTGTTACTTGCTATGCTCTCTGCAAACTCAATGGCACAGCCAGAAGGCTCTGCAGACCCAGATGCGATGATAGAACGAGAAGTTCGGGGAGTATGGAGTACTATTCTGGGTGGTTTAAAAAAATTTGCAAAAGTTCATGCCAAGAAAGTGTTTCCATTACATTGAAAACGCAGTGCTGATAAATAAAGATTTTCCAGGTGGGTTGGTTGAATGCACTTTGGACATCTCAGGCCATCTCTCCCTAAACCTGTAAAGATAAACAACAAATGCTGAAAAATAAAATGATATGTAGA

>Silurana tropicalis xpf-St7

AGAGCAAAAACTCTGAACAGACAACCTTGCCGCTTCTGTAACAGAAAGAGACCTCCATTTGGAAAGAATGTTGAAAGGAATATTCCTCTGTGTATTACTTGCTGTGCTCTCTGCAAACTCAATGGCACAGCCAGTAGGCTCTGCAGACCCAGATGAGATGATTGAACGAGAAGTTCGGGGACTCCTTTCTAATGTCGCAGGTCTTTTGAAACAATTTGCAAAAGGTGGTGTAAATGCTGTATTGAACCCAAAACGAGAAGCAATGTCTGTCAACAATGATGGCTTCAATTCTGTTCATTCCAAGAGATTTATTGGGGCACTTCTTGGTCCATTGTTGAATTTGCTTAAGGGAAGATAACTAATTGAAGACATTCGCTGGTAAGTATGGTAAATGAATAAAATGAGCAGACTTATCTTGAAAGTGACAGTGCTGAAGATTCTTCAGACCTAAAAAATATACCCCTCTTCCTGTCCAGAGAAAATAAAATAAATATCAGCCAAACTTG

>Silurana tropicalis xpf-St8

AGAGCAAAATATCTGAACAGACAACCTTGCCGCTTCTGTAACAGAAAGAGACCTCCATTTGGAAAGAATGTTGAAAGGAATATTCCTCTGTGTATTACTTGCTGTGCTCTCTGCAAACTCAATGGCACAGCCAGTAGGCTCTGCAGATGCAGATGAGATGATTGAACGAGAAGTTCGGGGATTCATGTCTAAAGTTGCAAATTTTGCCAAAAAATTTGCAAAAGGTGGTGTAAATGCTATAATGAATCAAAAACGAGAAGCAATGCCTGTCAACAGTGATGACTTCAATTCTGCTCATTCCAAGAGATTTATTGGGGCGCTTCTTCGTCCAGCGTTGAAGTGTGGTAAATGAATAAAATGGGCAGATTTATCTTGAGTCACAGTGCTGAAGATCCTTTTTTGAGACACTTCAGACCTACATCATATACCCCTTTTCATGTCCAGAGGAAATAAAATAAAATATATATCAGC

>Silurana tropicalis pgla-St2

GGAGTACAACAATTTGCATTGTGCACGTTGTTTCTTCTGGTATTGAGAACTGAATACATTTGAAAGGATGTACAAAGGGATATTCCTCTGCGTATTCCTTGCTGCAATCTGTGCAAATGCACTGGCACAGCCCACAGGCTTTGCAGATGCAGATGAAGAAAGCCACGAGCGATACATCCGAGGAATGGCAACTAAAGCTGGAACGGCGTTTGGAAAAGCTGCAAAAGCTATTATTGGTGCTGCTCTGGGACGTCGCAGTGCTCTGGAACTTGAGGATCTCATTCCAGTTAAAAGAATGGCTCAAATTGATTAAACCAGGAAATTAAATGGCCAGGTCGAATTTTTGCTCTACTGAAGACTAATATGAATGTCTGCAATGCGCCAAACCTATAAAAATATACTTCTGTTGTGAAATGCTGAAAATAAAACGACCTATATGCATGCAAAAAAAAAAAAAA

>Silurana tropicalis pgla-St3

AGTACAGCCATTTCCATTGAGCACTTTGTTTCTTCTGGTATTGAGGACTGAATACATTTGAAAGGATGTACAAAGGGATATTCCTCTGCGTATTCCTTGCTGCAATCTGTGCAAATGCACTGGCACAGCCCACAGGCTTTGCAGATGCAGATGAAGAAAGCCACGAGCGGTACATCCGAGGAATGGCAACTAAAGCTGGAACGGTGTTGGGAAAAGTTACAAAAGCTATTATTGGTGCTGCTCTGGGACGTCGCAGTGCTCGGGACCTTCAGGATCTCATTCCAGCTAAAAGAATGGCTCTATTGGACCTTGAAAAAAAAATTAAGCAATTTCTCCCAGATTAAACCAGGAATTTAAACGGCCAGGTCGGATTTTTGCTCTACTGAAGACTAACATGAATGTCTGCAATGCACCAAACCTAAAAAAATATACTTCTCTTGTGAAACACAGGAAATAAAGCGACCTATATGCATG
