# Supplementary material for: Origin and Functional Diversification of an Amphibian Defense Peptide Arsenal
Source: PLoS Genet. 2013 Aug 1;9(8):e1003662. doi: 10.1371/journal.pgen.1003662 (PMC3731216; doi:10.1371/journal.pgen.1003662)
Supplement: Text S2 — Results of an MS/MS ion search against a database of Silurana tropicalis AMP/HLP precursor proteins predicted from cDNA and gene sequences, using an in-house version of MASCOT Server (Matrix Science, USA). Individual ions scores >26 indicate identity or extensive homology (p<0.05). For each of the confirmed peptides, the MS/MS fragmentation spectrum and corresponding table with fragmentation masses is shown. a, C-terminal amidation; Mr, molecular weight; pQ, pyroglutamate. (DOC) [file pgen.1003662.s002.doc]

Supporting information: Text S2.

Results of an MS/MS ion search against a database of *Silurana tropicalis* AMP/HLP precursor proteins predicted from cDNA and gene sequences, using an in-house version of MASCOT Server (Matrix Science, USA) . Individual ions scores > 26 indicate identity or extensive homology (p < 0.05). For each of the confirmed peptides, the MS/MS fragmentation spectrum and corresponding table with fragmentation masses is shown. a, C-terminal amidation; Mr, molecular weight; pQ, pyroglutamate

1. CPF-St4

|  | **Observed Mr** | **Expected Mr** | **Calculated Mr** | **Delta** | **Score** | **Peptide** |
| --- | --- | --- | --- | --- | --- | --- |
| **CPF-St4** | 980.6714 | 2938.9925 | 2938.6579 | 0.3346 | 86 | R.SLFGTFAKMALKGASKLIPHLLPSRQQ.R |

**
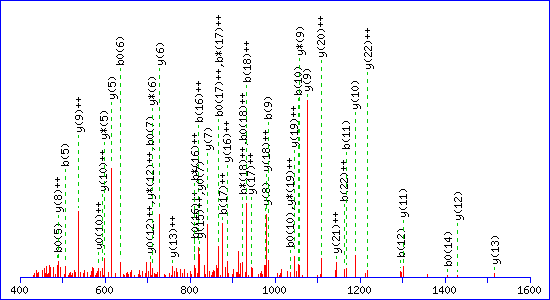
**

| **#** | **b** | **b^++^** | **b*** | **b*^++^** | **b^0^** | **b^0++^** | **Seq.** | **y** | **y^++^** | **y*** | **y*^++^** | **y^0^** | **y^0++^** | **#** |
| --- | --- | --- | --- | --- | --- | --- | --- | --- | --- | --- | --- | --- | --- | --- |
| **1** | 88.0393 | 44.5233 |  |  | 70.0287 | 35.5180 | **S** |  |  |  |  |  |  | **27** |
| **2** | 201.1234 | 101.0653 |  |  | 183.1128 | 92.0600 | **L** | 2852.6331 | 1426.8202 | 2835.6066 | 1418.3069 | 2834.6226 | 1417.8149 | **26** |
| **3** | 348.1918 | 174.5995 |  |  | 330.1812 | 165.5942 | **F** | 2739.5491 | 1370.2782 | 2722.5225 | 1361.7649 | 2721.5385 | 1361.2729 | **25** |
| **4** | 405.2132 | 203.1103 |  |  | 387.2027 | 194.1050 | **G** | 2592.4807 | 1296.7440 | 2575.4541 | 1288.2307 | 2574.4701 | 1287.7387 | **24** |
| **5** | **506.2609** | 253.6341 |  |  | **488.2504** | 244.6288 | **T** | 2535.4592 | 1268.2332 | 2518.4326 | 1259.7200 | 2517.4486 | 1259.2280 | **23** |
| **6** | 653.3293 | 327.1683 |  |  | **635.3188** | 318.1630 | **F** | 2434.4115 | **1217.7094** | 2417.3850 | 1209.1961 | 2416.4010 | 1208.7041 | **22** |
| **7** | 724.3665 | 362.6869 |  |  | **706.3559** | 353.6816 | **A** | 2287.3431 | **1144.1752** | 2270.3166 | 1135.6619 | 2269.3325 | 1135.1699 | **21** |
| **8** | 852.4614 | 426.7343 | 835.4349 | 418.2211 | 834.4509 | 417.7291 | **K** | 2216.3060 | **1108.6566** | 2199.2794 | 1100.1434 | 2198.2954 | 1099.6514 | **20** |
| **9** | **983.5019** | 492.2546 | 966.4754 | 483.7413 | 965.4913 | 483.2493 | **M** | 2088.2110 | **1044.6092** | 2071.1845 | **1036.0959** | 2070.2005 | 1035.6039 | **19** |
| **10** | **1054.5390** | 527.7731 | 1037.5125 | 519.2599 | **1036.5285** | 518.7679 | **A** | 1957.1705 | **979.0889** | 1940.1440 | 970.5756 | 1939.1600 | 970.0836 | **18** |
| **11** | **1167.6231** | 584.3152 | 1150.5965 | 575.8019 | 1149.6125 | 575.3099 | **L** | 1886.1334 | **943.5704** | 1869.1069 | 935.0571 | 1868.1229 | 934.5651 | **17** |
| **12** | **1295.7180** | 648.3627 | 1278.6915 | 639.8494 | 1277.7075 | 639.3574 | **K** | 1773.0494 | **887.0283** | 1756.0228 | 878.5150 | 1755.0388 | 878.0230 | **16** |
| **13** | 1352.7395 | 676.8734 | 1335.7130 | 668.3601 | 1334.7289 | 667.8681 | **G** | 1644.9544 | **822.9808** | 1627.9279 | 814.4676 | 1626.9438 | 813.9756 | **15** |
| **14** | 1423.7766 | 712.3919 | 1406.7501 | 703.8787 | **1405.7661** | 703.3867 | **A** | 1587.9329 | 794.4701 | 1570.9064 | 785.9568 | 1569.9224 | 785.4648 | **14** |
| **15** | 1510.8086 | 755.9080 | 1493.7821 | 747.3947 | 1492.7981 | 746.9027 | **S** | **1516.8958** | **758.9515** | 1499.8693 | 750.4383 | 1498.8853 | 749.9463 | **13** |
| **16** | 1638.9036 | **819.9554** | 1621.8771 | **811.4422** | 1620.8930 | **810.9502** | **K** | **1429.8638** | 715.4355 | 1412.8372 | **706.9223** | 1411.8532 | **706.4303** | **12** |
| **17** | 1751.9877 | **876.4975** | 1734.9611 | **867.9842** | 1733.9771 | **867.4922** | **L** | **1301.7688** | 651.3881 | 1284.7423 | 642.8748 | 1283.7583 | 642.3828 | **11** |
| **18** | 1865.0717 | **933.0395** | 1848.0452 | **924.5262** | 1847.0612 | **924.0342** | **I** | **1188.6848** | **594.8460** | 1171.6582 | 586.3327 | 1170.6742 | **585.8407** | **10** |
| **19** | 1962.1245 | 981.5659 | 1945.0980 | 973.0526 | 1944.1139 | 972.5606 | **P** | **1075.6007** | **538.3040** | **1058.5742** | 529.7907 | 1057.5901 | 529.2987 | **9** |
| **20** | 2099.1834 | 1050.0953 | 2082.1569 | 1041.5821 | 2081.1728 | 1041.0901 | **H** | **978.5479** | **489.7776** | 961.5214 | 481.2643 | 960.5374 | 480.7723 | **8** |
| **21** | 2212.2675 | 1106.6374 | 2195.2409 | 1098.1241 | 2194.2569 | 1097.6321 | **L** | **841.4890** | 421.2482 | 824.4625 | 412.7349 | **823.4785** | 412.2429 | **7** |
| **22** | 2325.3515 | **1163.1794** | 2308.3250 | 1154.6661 | 2307.3410 | 1154.1741 | **L** | **728.4050** | 364.7061 | **711.3784** | 356.1928 | 710.3944 | 355.7008 | **6** |
| **23** | 2422.4043 | 1211.7058 | 2405.3778 | 1203.1925 | 2404.3937 | 1202.7005 | **P** | **615.3209** | 308.1641 | **598.2944** | 299.6508 | 597.3103 | 299.1588 | **5** |
| **24** | 2509.4363 | 1255.2218 | 2492.4098 | 1246.7085 | 2491.4258 | 1246.2165 | **S** | 518.2681 | 259.6377 | 501.2416 | 251.1244 | 500.2576 | 250.6324 | **4** |
| **25** | 2665.5374 | 1333.2724 | 2648.5109 | 1324.7591 | 2647.5269 | 1324.2671 | **R** | 431.2361 | 216.1217 | 414.2096 | 207.6084 |  |  | **3** |
| **26** | 2793.5960 | 1397.3016 | 2776.5695 | 1388.7884 | 2775.5855 | 1388.2964 | **Q** | 275.1350 | 138.0711 | 258.1084 | 129.5579 |  |  | **2** |
| **27** |  |  |  |  |  |  | **Q** | 147.0764 | 74.0418 | 130.0499 | 65.5286 |  |  | **1** |

2. CPF-St5

|  | **Observed Mr** | **Expected Mr** | **Calculated Mr** | **Delta** | **Score** | **Peptide** |
| --- | --- | --- | --- | --- | --- | --- |
| **CPF-St5** | 938.6693 | 2812.9862 | 2812.6803 | 0.3059 | 96 | R.GVFGLLAKAALKGASKLIPHLLPSRQQ.R |

**
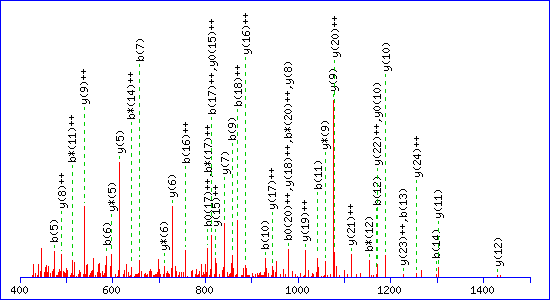
**

| **#** | **b** | **b^++^** | **b*** | **b*^++^** | **b^0^** | **b^0++^** | **Seq.** | **y** | **y^++^** | **y*** | **y*^++^** | **y^0^** | **y^0++^** | **#** |
| --- | --- | --- | --- | --- | --- | --- | --- | --- | --- | --- | --- | --- | --- | --- |
| **1** | 58.0287 | 29.5180 |  |  |  |  | **G** |  |  |  |  |  |  | **27** |
| **2** | 157.0972 | 79.0522 |  |  |  |  | **V** | 2756.6662 | 1378.8367 | 2739.6396 | 1370.3234 | 2738.6556 | 1369.8314 | **26** |
| **3** | 304.1656 | 152.5864 |  |  |  |  | **F** | 2657.5977 | 1329.3025 | 2640.5712 | 1320.7892 | 2639.5872 | 1320.2972 | **25** |
| **4** | 361.1870 | 181.0972 |  |  |  |  | **G** | 2510.5293 | **1255.7683** | 2493.5028 | 1247.2550 | 2492.5188 | 1246.7630 | **24** |
| **5** | **474.2711** | 237.6392 |  |  |  |  | **L** | 2453.5079 | **1227.2576** | 2436.4813 | 1218.7443 | 2435.4973 | 1218.2523 | **23** |
| **6** | **587.3552** | 294.1812 |  |  |  |  | **L** | 2340.4238 | **1170.7155** | 2323.3972 | 1162.2023 | 2322.4132 | 1161.7103 | **22** |
| **7** | **658.3923** | 329.6998 |  |  |  |  | **A** | 2227.3397 | **1114.1735** | 2210.3132 | 1105.6602 | 2209.3292 | 1105.1682 | **21** |
| **8** | 786.4872 | 393.7473 | 769.4607 | 385.2340 |  |  | **K** | 2156.3026 | **1078.6549** | 2139.2761 | 1070.1417 | 2138.2921 | 1069.6497 | **20** |
| **9** | **857.5244** | 429.2658 | 840.4978 | 420.7525 |  |  | **A** | 2028.2077 | **1014.6075** | 2011.1811 | 1006.0942 | 2010.1971 | 1005.6022 | **19** |
| **10** | **928.5615** | 464.7844 | 911.5349 | 456.2711 |  |  | **A** | 1957.1705 | **979.0889** | 1940.1440 | 970.5756 | 1939.1600 | 970.0836 | **18** |
| **11** | **1041.6455** | 521.3264 | 1024.6190 | **512.8131** |  |  | **L** | 1886.1334 | **943.5704** | 1869.1069 | 935.0571 | 1868.1229 | 934.5651 | **17** |
| **12** | **1169.7405** | 585.3739 | **1152.7139** | 576.8606 |  |  | **K** | 1773.0494 | **887.0283** | 1756.0228 | 878.5150 | 1755.0388 | 878.0230 | **16** |
| **13** | **1226.7620** | 613.8846 | 1209.7354 | 605.3713 |  |  | **G** | 1644.9544 | **822.9808** | 1627.9279 | 814.4676 | 1626.9438 | **813.9756** | **15** |
| **14** | **1297.7991** | 649.4032 | 1280.7725 | **640.8899** |  |  | **A** | 1587.9329 | 794.4701 | 1570.9064 | 785.9568 | 1569.9224 | 785.4648 | **14** |
| **15** | 1384.8311 | 692.9192 | 1367.8045 | 684.4059 | 1366.8205 | 683.9139 | **S** | 1516.8958 | 758.9515 | 1499.8693 | 750.4383 | 1498.8853 | 749.9463 | **13** |
| **16** | 1512.9261 | **756.9667** | 1495.8995 | 748.4534 | 1494.9155 | 747.9614 | **K** | **1429.8638** | 715.4355 | 1412.8372 | 706.9223 | 1411.8532 | 706.4303 | **12** |
| **17** | 1626.0101 | **813.5087** | 1608.9836 | **804.9954** | 1607.9996 | **804.5034** | **L** | **1301.7688** | 651.3881 | 1284.7423 | 642.8748 | 1283.7583 | 642.3828 | **11** |
| **18** | 1739.0942 | **870.0507** | 1722.0676 | 861.5375 | 1721.0836 | 861.0454 | **I** | **1188.6848** | 594.8460 | 1171.6582 | 586.3327 | **1170.6742** | 585.8407 | **10** |
| **19** | 1836.1470 | 918.5771 | 1819.1204 | 910.0638 | 1818.1364 | 909.5718 | **P** | **1075.6007** | **538.3040** | **1058.5742** | 529.7907 | 1057.5901 | 529.2987 | **9** |
| **20** | 1973.2059 | 987.1066 | 1956.1793 | **978.5933** | 1955.1953 | **978.1013** | **H** | **978.5479** | **489.7776** | 961.5214 | 481.2643 | 960.5374 | 480.7723 | **8** |
| **21** | 2086.2899 | 1043.6486 | 2069.2634 | 1035.1353 | 2068.2794 | 1034.6433 | **L** | **841.4890** | 421.2482 | 824.4625 | 412.7349 | 823.4785 | 412.2429 | **7** |
| **22** | 2199.3740 | 1100.1906 | 2182.3474 | 1091.6774 | 2181.3634 | 1091.1854 | **L** | **728.4050** | 364.7061 | **711.3784** | 356.1928 | 710.3944 | 355.7008 | **6** |
| **23** | 2296.4268 | 1148.7170 | 2279.4002 | 1140.2037 | 2278.4162 | 1139.7117 | **P** | **615.3209** | 308.1641 | **598.2944** | 299.6508 | 597.3103 | 299.1588 | **5** |
| **24** | 2383.4588 | 1192.2330 | 2366.4322 | 1183.7198 | 2365.4482 | 1183.2277 | **S** | 518.2681 | 259.6377 | 501.2416 | 251.1244 | 500.2576 | 250.6324 | **4** |
| **25** | 2539.5599 | 1270.2836 | 2522.5333 | 1261.7703 | 2521.5493 | 1261.2783 | **R** | 431.2361 | 216.1217 | 414.2096 | 207.6084 |  |  | **3** |
| **26** | 2667.6185 | 1334.3129 | 2650.5919 | 1325.7996 | 2649.6079 | 1325.3076 | **Q** | 275.1350 | 138.0711 | 258.1084 | 129.5579 |  |  | **2** |
| **27** |  |  |  |  |  |  | **Q** | 147.0764 | 74.0418 | 130.0499 | 65.5286 |  |  | **1** |

3. CPF-St7

|  | **Observed Mr** | **Expected Mr** | **Calculated Mr** | **Delta** | **Score** | **Peptide** |
| --- | --- | --- | --- | --- | --- | --- |
| **CPF-St7** | 920.1692 | 1838.3239 | 1838.1353 | 0.1886 | 30 | R.NLLGSLLKTGLKVGSNLLa.(G)R |


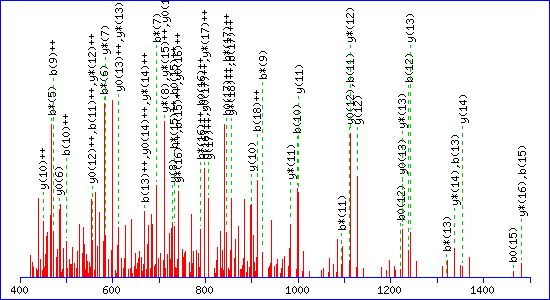


| **#** | **b** | **b^++^** | **b*** | **b*^++^** | **b^0^** | **b^0++^** | **Seq.** | **y** | **y^++^** | **y*** | **y*^++^** | **y^0^** | **y^0++^** | **#** |
| --- | --- | --- | --- | --- | --- | --- | --- | --- | --- | --- | --- | --- | --- | --- |
| **1** | 115.0502 | 58.0287 | 98.0237 | 49.5155 |  |  | **N** |  |  |  |  |  |  | **19** |
| **2** | 228.1343 | 114.5708 | 211.1077 | 106.0575 |  |  | **L** | 1725.0997 | 863.0535 | 1708.0731 | **854.5402** | 1707.0891 | 854.0482 | **18** |
| **3** | 341.2183 | 171.1128 | 324.1918 | 162.5995 |  |  | **L** | 1612.0156 | **806.5114** | 1594.9891 | **797.9982** | 1594.0050 | **797.5062** | **17** |
| **4** | 398.2398 | 199.6235 | 381.2132 | 191.1103 |  |  | **G** | 1498.9315 | 749.9694 | **1481.9050** | **741.4561** | 1480.9210 | **740.9641** | **16** |
| **5** | 485.2718 | 243.1395 | **468.2453** | 234.6263 | 467.2613 | 234.1343 | **S** | 1441.9101 | 721.4587 | 1424.8835 | **712.9454** | 1423.8995 | **712.4534** | **15** |
| **6** | 598.3559 | 299.6816 | **581.3293** | 291.1683 | 580.3453 | 290.6763 | **L** | **1354.8780** | 677.9427 | **1337.8515** | **669.4294** | 1336.8675 | **668.9374** | **14** |
| **7** | 711.4400 | 356.2236 | **694.4134** | 347.7103 | 693.4294 | 347.2183 | **L** | **1241.7940** | 621.4006 | **1224.7674** | **612.8874** | **1223.7834** | **612.3953** | **13** |
| **8** | 839.5349 | 420.2711 | 822.5084 | 411.7578 | 821.5243 | 411.2658 | **K** | **1128.7099** | 564.8586 | **1111.6834** | **556.3453** | **1110.6994** | **555.8533** | **12** |
| **9** | 940.5826 | **470.7949** | **923.5560** | 462.2817 | 922.5720 | 461.7897 | **T** | **1000.6150** | 500.8111 | **983.5884** | 492.2978 | 982.6044 | 491.8058 | **11** |
| **10** | **997.6041** | **499.3057** | 980.5775 | 490.7924 | 979.5935 | 490.3004 | **G** | **899.5673** | **450.2873** | 882.5407 | 441.7740 | 881.5567 | 441.2820 | **10** |
| **11** | **1110.6881** | **555.8477** | **1093.6616** | 547.3344 | 1092.6776 | 546.8424 | **L** | 842.5458 | 421.7765 | 825.5193 | 413.2633 | 824.5352 | 412.7713 | **9** |
| **12** | **1238.7831** | 619.8952 | 1221.7565 | 611.3819 | **1220.7725** | 610.8899 | **K** | **729.4618** | 365.2345 | **712.4352** | 356.7212 | 711.4512 | 356.2292 | **8** |
| **13** | **1337.8515** | **669.4294** | **1320.8249** | 660.9161 | 1319.8409 | 660.4241 | **V** | 601.3668 | 301.1870 | **584.3402** | 292.6738 | 583.3562 | 292.1817 | **7** |
| **14** | 1394.8730 | 697.9401 | 1377.8464 | 689.4268 | 1376.8624 | 688.9348 | **G** | 502.2984 | 251.6528 | 485.2718 | 243.1396 | **484.2878** | 242.6475 | **6** |
| **15** | **1481.9050** | **741.4561** | 1464.8784 | **732.9429** | **1463.8944** | **732.4509** | **S** | 445.2769 | 223.1421 | 428.2504 | 214.6288 | 427.2663 | 214.1368 | **5** |
| **16** | 1595.9479 | **798.4776** | 1578.9214 | **789.9643** | 1577.9374 | **789.4723** | **N** | 358.2449 | 179.6261 | 341.2183 | 171.1128 |  |  | **4** |
| **17** | 1709.0320 | **855.0196** | 1692.0054 | **846.5064** | 1691.0214 | **846.0143** | **L** | 244.2020 | 122.6046 |  |  |  |  | **3** |
| **18** | 1822.1160 | **911.5617** | 1805.0895 | 903.0484 | 1804.1055 | 902.5564 | **L** | 131.1179 | 66.0626 |  |  |  |  | **2** |
| **19** |  |  |  |  |  |  | **G** | 18.0338 | 9.5206 |  |  |  |  | **1** |

4. Magainin-St1

|  | **Observed Mr** | **Expected Mr** | **Calculated Mr** | **Delta** | **Score** | **Peptide** |
| --- | --- | --- | --- | --- | --- | --- |
| **magainin-St1** | 1167.2810 | 2332.5473 | 2332.2903 | 0.2571 | 112 | R.GLKEVAHSAKKFAKGFISGLTGS.K |


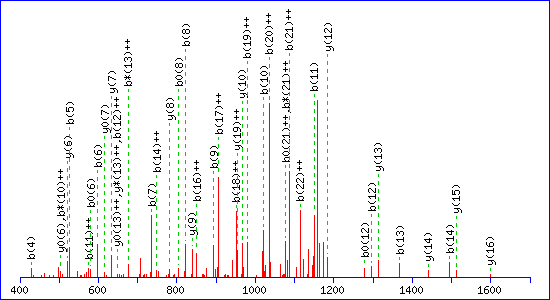


| **#** | **b** | **b^++^** | **b*** | **b*^++^** | **b^0^** | **b^0++^** | **Seq.** | **y** | **y^++^** | **y*** | **y*^++^** | **y^0^** | **y^0++^** | **#** |
| --- | --- | --- | --- | --- | --- | --- | --- | --- | --- | --- | --- | --- | --- | --- |
| **1** | 58.0287 | 29.5180 |  |  |  |  | **G** |  |  |  |  |  |  | **23** |
| **2** | 171.1128 | 86.0600 |  |  |  |  | **L** | 2276.2761 | 1138.6417 | 2259.2496 | 1130.1284 | 2258.2656 | 1129.6364 | **22** |
| **3** | 299.2078 | 150.1075 | 282.1812 | 141.5942 |  |  | **K** | 2163.1921 | 1082.0997 | 2146.1655 | 1073.5864 | 2145.1815 | 1073.0944 | **21** |
| **4** | **428.2504** | 214.6288 | 411.2238 | 206.1155 | 410.2398 | 205.6235 | **E** | 2035.0971 | 1018.0522 | 2018.0706 | 1009.5389 | 2017.0865 | 1009.0469 | **20** |
| **5** | **527.3188** | 264.1630 | 510.2922 | 255.6498 | 509.3082 | 255.1577 | **V** | 1906.0545 | **953.5309** | 1889.0280 | 945.0176 | 1888.0439 | 944.5256 | **19** |
| **6** | **598.3559** | 299.6816 | 581.3293 | 291.1683 | **580.3453** | 290.6763 | **A** | 1806.9861 | 903.9967 | 1789.9595 | 895.4834 | 1788.9755 | 894.9914 | **18** |
| **7** | **735.4148** | 368.2110 | 718.3883 | 359.6978 | 717.4042 | 359.2058 | **H** | 1735.9490 | 868.4781 | 1718.9224 | 859.9649 | 1717.9384 | 859.4728 | **17** |
| **8** | **822.4468** | 411.7271 | 805.4203 | 403.2138 | **804.4363** | 402.7218 | **S** | **1598.8901** | 799.9487 | 1581.8635 | 791.4354 | 1580.8795 | 790.9434 | **16** |
| **9** | **893.4839** | 447.2456 | 876.4574 | 438.7323 | 875.4734 | 438.2403 | **A** | **1511.8580** | 756.4327 | 1494.8315 | 747.9194 | 1493.8475 | 747.4274 | **15** |
| **10** | **1021.5789** | 511.2931 | 1004.5524 | **502.7798** | 1003.5683 | 502.2878 | **K** | **1440.8209** | 720.9141 | 1423.7944 | 712.4008 | 1422.8104 | 711.9088 | **14** |
| **11** | **1149.6739** | **575.3406** | 1132.6473 | 566.8273 | 1131.6633 | 566.3353 | **K** | **1312.7260** | 656.8666 | 1295.6994 | **648.3533** | 1294.7154 | **647.8613** | **13** |
| **12** | **1296.7423** | **648.8748** | 1279.7157 | 640.3615 | **1278.7317** | 639.8695 | **F** | **1184.6310** | 592.8191 | 1167.6045 | 584.3059 | 1166.6204 | 583.8139 | **12** |
| **13** | **1367.7794** | 684.3933 | 1350.7528 | **675.8801** | 1349.7688 | 675.3881 | **A** | 1037.5626 | 519.2849 | 1020.5360 | 510.7717 | 1019.5520 | 510.2796 | **11** |
| **14** | **1495.8744** | **748.4408** | 1478.8478 | 739.9275 | 1477.8638 | 739.4355 | **K** | **966.5255** | 483.7664 | 949.4989 | 475.2531 | 948.5149 | 474.7611 | **10** |
| **15** | 1552.8958 | 776.9515 | 1535.8693 | 768.4383 | 1534.8853 | 767.9463 | **G** | **838.4305** | 419.7189 |  |  | 820.4199 | 410.7136 | **9** |
| **16** | 1699.9642 | **850.4858** | 1682.9377 | 841.9725 | 1681.9537 | 841.4805 | **F** | **781.4090** | 391.2082 |  |  | 763.3985 | 382.2029 | **8** |
| **17** | 1813.0483 | **907.0278** | 1796.0218 | 898.5145 | 1795.0377 | 898.0225 | **I** | **634.3406** | 317.6740 |  |  | **616.3301** | 308.6687 | **7** |
| **18** | 1900.0803 | **950.5438** | 1883.0538 | 942.0305 | 1882.0698 | 941.5385 | **S** | **521.2566** | 261.1319 |  |  | **503.2460** | 252.1266 | **6** |
| **19** | 1957.1018 | **979.0545** | 1940.0752 | 970.5413 | 1939.0912 | 970.0493 | **G** | 434.2245 | 217.6159 |  |  | 416.2140 | 208.6106 | **5** |
| **20** | 2070.1859 | **1035.5966** | 2053.1593 | 1027.0833 | 2052.1753 | 1026.5913 | **L** | 377.2031 | 189.1052 |  |  | 359.1925 | 180.0999 | **4** |
| **21** | 2171.2335 | **1086.1204** | 2154.2070 | **1077.6071** | 2153.2230 | **1077.1151** | **T** | 264.1190 | 132.5631 |  |  | 246.1084 | 123.5579 | **3** |
| **22** | 2228.2550 | **1114.6311** | 2211.2285 | 1106.1179 | 2210.2444 | 1105.6259 | **G** | 163.0713 | 82.0393 |  |  | 145.0608 | 73.0340 | **2** |
| **23** |  |  |  |  |  |  | **S** | 106.0499 | 53.5286 |  |  | 88.0393 | 44.5233 | **1** |

5. XPF-St4

|  | **Observed Mr** | **Expected Mr** | **Calculated Mr** | **Delta** | **Score** | **Peptide** |
| --- | --- | --- | --- | --- | --- | --- |
| **XPF-St4** | 1260.3378 | 2518.6611 | 2518.3696 | 0.2915 | 82 | R.GWASSIGSILGKFAKGGAQAFLQPK.R |


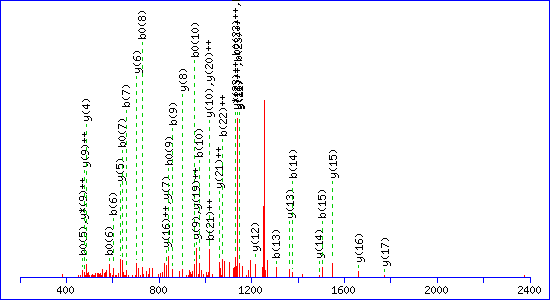


| **#** | **b** | **b^++^** | **b*** | **b*^++^** | **b^0^** | **b^0++^** | **Seq.** | **y** | **y^++^** | **y*** | **y*^++^** | **y^0^** | **y^0++^** | **#** |
| --- | --- | --- | --- | --- | --- | --- | --- | --- | --- | --- | --- | --- | --- | --- |
| **1** | 58.0287 | 29.5180 |  |  |  |  | **G** |  |  |  |  |  |  | **25** |
| **2** | 244.1081 | 122.5577 |  |  |  |  | **W** | 2462.3554 | 1231.6814 | 2445.3289 | 1223.1681 | 2444.3449 | 1222.6761 | **24** |
| **3** | 315.1452 | 158.0762 |  |  |  |  | **A** | 2276.2761 | **1138.6417** | 2259.2496 | **1130.1284** | 2258.2656 | **1129.6364** | **23** |
| **4** | 402.1772 | 201.5922 |  |  | 384.1666 | 192.5870 | **S** | 2205.2390 | 1103.1231 | 2188.2125 | 1094.6099 | 2187.2285 | 1094.1179 | **22** |
| **5** | 489.2092 | 245.1082 |  |  | **471.1987** | 236.1030 | **S** | 2118.2070 | **1059.6071** | 2101.1804 | 1051.0939 | 2100.1964 | 1050.6018 | **21** |
| **6** | **602.2933** | 301.6503 |  |  | **584.2827** | 292.6450 | **I** | 2031.1750 | **1016.0911** | 2014.1484 | 1007.5778 | 2013.1644 | 1007.0858 | **20** |
| **7** | **659.3148** | 330.1610 |  |  | **641.3042** | 321.1557 | **G** | 1918.0909 | **959.5491** | 1901.0643 | 951.0358 | 1900.0803 | 950.5438 | **19** |
| **8** | 746.3468 | 373.6770 |  |  | **728.3362** | 364.6717 | **S** | 1861.0694 | 931.0384 | 1844.0429 | 922.5251 | 1843.0589 | 922.0331 | **18** |
| **9** | **859.4308** | 430.2191 |  |  | **841.4203** | 421.2138 | **I** | **1774.0374** | 887.5223 | 1757.0109 | 879.0091 |  |  | **17** |
| **10** | **972.5149** | 486.7611 |  |  | **954.5043** | 477.7558 | **L** | **1660.9533** | **830.9803** | 1643.9268 | 822.4670 |  |  | **16** |
| **11** | 1029.5364 | 515.2718 |  |  | 1011.5258 | 506.2665 | **G** | **1547.8693** | 774.4383 | 1530.8427 | 765.9250 |  |  | **15** |
| **12** | 1157.6313 | 579.3193 | 1140.6048 | 570.8060 | 1139.6208 | 570.3140 | **K** | **1490.8478** | 745.9275 | 1473.8213 | 737.4143 |  |  | **14** |
| **13** | **1304.6997** | 652.8535 | 1287.6732 | 644.3402 | 1286.6892 | 643.8482 | **F** | **1362.7528** | 681.8801 | 1345.7263 | 673.3668 |  |  | **13** |
| **14** | **1375.7369** | 688.3721 | 1358.7103 | 679.8588 | 1357.7263 | 679.3668 | **A** | **1215.6844** | 608.3459 | 1198.6579 | 599.8326 |  |  | **12** |
| **15** | **1503.8318** | 752.4196 | 1486.8053 | 743.9063 | 1485.8213 | 743.4143 | **K** | **1144.6473** | 572.8273 | 1127.6208 | 564.3140 |  |  | **11** |
| **16** | 1560.8533 | 780.9303 | 1543.8267 | 772.4170 | 1542.8427 | 771.9250 | **G** | **1016.5524** | 508.7798 | 999.5258 | 500.2665 |  |  | **10** |
| **17** | 1617.8748 | 809.4410 | 1600.8482 | 800.9277 | 1599.8642 | 800.4357 | **G** | **959.5309** | **480.2691** | 942.5043 | **471.7558** |  |  | **9** |
| **18** | 1688.9119 | 844.9596 | 1671.8853 | 836.4463 | 1670.9013 | 835.9543 | **A** | **902.5094** | 451.7584 | 885.4829 | 443.2451 |  |  | **8** |
| **19** | 1816.9704 | 908.9889 | 1799.9439 | 900.4756 | 1798.9599 | 899.9836 | **Q** | **831.4723** | 416.2398 | 814.4458 | 407.7265 |  |  | **7** |
| **20** | 1888.0076 | 944.5074 | 1870.9810 | 935.9941 | 1869.9970 | 935.5021 | **A** | **703.4137** | 352.2105 | 686.3872 | 343.6972 |  |  | **6** |
| **21** | 2035.0760 | **1018.0416** | 2018.0494 | 1009.5284 | 2017.0654 | 1009.0363 | **F** | **632.3766** | 316.6920 | 615.3501 | 308.1787 |  |  | **5** |
| **22** | 2148.1600 | **1074.5837** | 2131.1335 | 1066.0704 | 2130.1495 | 1065.5784 | **L** | **485.3082** | 243.1577 | 468.2817 | 234.6445 |  |  | **4** |
| **23** | 2276.2186 | **1138.6129** | 2259.1921 | **1130.0997** | 2258.2081 | **1129.6077** | **Q** | 372.2241 | 186.6157 | 355.1976 | 178.1024 |  |  | **3** |
| **24** | 2373.2714 | 1187.1393 | 2356.2448 | 1178.6261 | 2355.2608 | 1178.1340 | **P** | 244.1656 | 122.5864 | 227.1390 | 114.0731 |  |  | **2** |
| **25** |  |  |  |  |  |  | **K** | 147.1128 | 74.0600 | 130.0863 | 65.5468 |  |  | **1** |

6. Levitide-St1

|  | **Observed Mr** | **Expected Mr** | **Calculated Mr** | **Delta** | **Score** | **Peptide** |
| --- | --- | --- | --- | --- | --- | --- |
| **Levitide-St1** | 773.5195 | 1545.0245 | 1543.8578 | 1.1667 | 43 | P.pQGLMGTLISKQMKKa.(G)- |


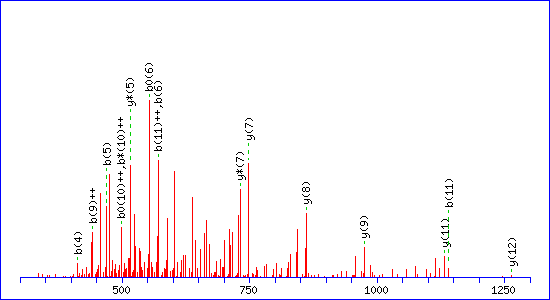


| **#** | **b** | **b^++^** | **b*** | **b*^++^** | **b^0^** | **b^0++^** | **Seq.** | **y** | **y^++^** | **y*** | **y*^++^** | **y^0^** | **y^0++^** | **#** |
| --- | --- | --- | --- | --- | --- | --- | --- | --- | --- | --- | --- | --- | --- | --- |
| **1** | 112.0393 | 56.5233 | 95.0128 | 48.0100 |  |  | **Q** |  |  |  |  |  |  | **15** |
| **2** | 169.0608 | 85.0340 | 152.0342 | 76.5208 |  |  | **G** | 1433.8331 | 717.4202 | 1416.8065 | 708.9069 | 1415.8225 | 708.4149 | **14** |
| **3** | 282.1448 | 141.5761 | 265.1183 | 133.0628 |  |  | **L** | 1376.8116 | 688.9095 | 1359.7851 | 680.3962 | 1358.8011 | 679.9042 | **13** |
| **4** | **413.1853** | 207.0963 | 396.1588 | 198.5830 |  |  | **M** | **1263.7276** | 632.3674 | 1246.7010 | 623.8541 | 1245.7170 | 623.3621 | **12** |
| **5** | **470.2068** | 235.6070 | 453.1802 | 227.0938 |  |  | **G** | **1132.6871** | 566.8472 | 1115.6605 | 558.3339 | 1114.6765 | 557.8419 | **11** |
| **6** | **571.2545** | 286.1309 | 554.2279 | 277.6176 | **553.2439** | 277.1256 | **T** | 1075.6656 | 538.3364 | 1058.6391 | 529.8232 | 1057.6550 | 529.3312 | **10** |
| **7** | 684.3385 | 342.6729 | 667.3120 | 334.1596 | 666.3280 | 333.6676 | **L** | **974.6179** | 487.8126 | 957.5914 | 479.2993 | 956.6074 | 478.8073 | **9** |
| **8** | 797.4226 | 399.2149 | 780.3961 | 390.7017 | 779.4120 | 390.2097 | **I** | **861.5339** | 431.2706 | 844.5073 | 422.7573 | 843.5233 | 422.2653 | **8** |
| **9** | 884.4546 | **442.7310** | 867.4281 | 434.2177 | 866.4441 | 433.7257 | **S** | **748.4498** | 374.7285 | **731.4233** | 366.2153 | 730.4392 | 365.7233 | **7** |
| **10** | 1012.5496 | 506.7784 | 995.5230 | **498.2652** | 994.5390 | **497.7732** | **K** | 661.4178 | 331.2125 | 644.3912 | 322.6993 |  |  | **6** |
| **11** | **1140.6082** | **570.8077** | 1123.5816 | 562.2944 | 1122.5976 | 561.8024 | **Q** | 533.3228 | 267.1650 | **516.2963** | 258.6518 |  |  | **5** |
| **12** | 1271.6487 | 636.3280 | 1254.6221 | 627.8147 | 1253.6381 | 627.3227 | **M** | 405.2642 | 203.1358 | 388.2377 | 194.6225 |  |  | **4** |
| **13** | 1399.7436 | 700.3754 | 1382.7171 | 691.8622 | 1381.7331 | 691.3702 | **K** | 274.2238 | 137.6155 | 257.1972 | 129.1022 |  |  | **3** |
| **14** | 1527.8386 | 764.4229 | 1510.8120 | 755.9097 | 1509.8280 | 755.4176 | **K** | 146.1288 | 73.5680 | 129.1022 | 65.0548 |  |  | **2** |
| **15** |  |  |  |  |  |  | **G** | 18.0338 | 9.5206 |  |  |  |  | **1** |

7. Levitide-St2

|  | **Observed Mr** | **Expected Mr** | **Calculated Mr** | **Delta** | **Score** | **Peptide** |
| --- | --- | --- | --- | --- | --- | --- |
| **Levitide-St2** | 741.0491 | 1480.0837 | 1479.9137 | 0.1700 | 7 | P.pQGLIGTLTAKQIKKa.(G)- |


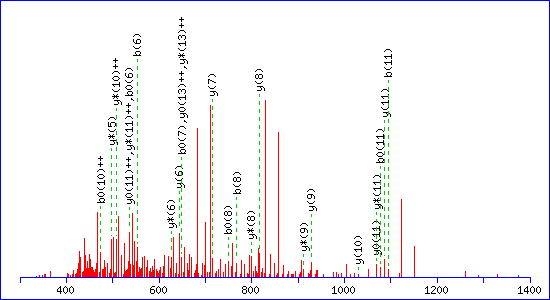


| **#** | **b** | **b^++^** | **b*** | **b*^++^** | **b^0^** | **b^0++^** | **Seq.** | **y** | **y^++^** | **y*** | **y*^++^** | **y^0^** | **y^0++^** | **#** |
| --- | --- | --- | --- | --- | --- | --- | --- | --- | --- | --- | --- | --- | --- | --- |
| **1** | 112.0393 | 56.5233 | 95.0128 | 48.0100 |  |  | **Q** |  |  |  |  |  |  | **15** |
| **2** | 169.0608 | 85.0340 | 152.0342 | 76.5208 |  |  | **G** | 1369.8890 | 685.4481 | 1352.8624 | 676.9348 | 1351.8784 | 676.4428 | **14** |
| **3** | 282.1448 | 141.5761 | 265.1183 | 133.0628 |  |  | **L** | 1312.8675 | 656.9374 | 1295.8409 | **648.4241** | 1294.8569 | **647.9321** | **13** |
| **4** | 395.2289 | 198.1181 | 378.2024 | 189.6048 |  |  | **I** | 1199.7834 | 600.3953 | 1182.7569 | 591.8821 | 1181.7729 | 591.3901 | **12** |
| **5** | 452.2504 | 226.6288 | 435.2238 | 218.1156 |  |  | **G** | **1086.6994** | 543.8533 | **1069.6728** | **535.3400** | **1068.6888** | **534.8480** | **11** |
| **6** | **553.2981** | 277.1527 | 536.2715 | 268.6394 | **535.2875** | 268.1474 | **T** | **1029.6779** | 515.3426 | 1012.6513 | **506.8293** | 1011.6673 | 506.3373 | **10** |
| **7** | 666.3821 | 333.6947 | 649.3556 | 325.1814 | **648.3716** | 324.6894 | **L** | **928.6302** | 464.8187 | **911.6037** | 456.3055 | 910.6196 | 455.8135 | **9** |
| **8** | **767.4298** | 384.2185 | 750.4032 | 375.7053 | **749.4192** | 375.2133 | **T** | **815.5462** | 408.2767 | **798.5196** | 399.7634 | 797.5356 | 399.2714 | **8** |
| **9** | 838.4669 | 419.7371 | 821.4404 | 411.2238 | 820.4563 | 410.7318 | **A** | **714.4985** | 357.7529 | 697.4719 | 349.2396 |  |  | **7** |
| **10** | 966.5619 | 483.7846 | 949.5353 | 475.2713 | 948.5513 | **474.7793** | **K** | **643.4614** | 322.2343 | **626.4348** | 313.7210 |  |  | **6** |
| **11** | **1094.6205** | 547.8139 | 1077.5939 | 539.3006 | **1076.6099** | 538.8086 | **Q** | 515.3664 | 258.1868 | **498.3398** | 249.6736 |  |  | **5** |
| **12** | 1207.7045 | 604.3559 | 1190.6780 | 595.8426 | 1189.6940 | 595.3506 | **I** | 387.3078 | 194.1575 | 370.2813 | 185.6443 |  |  | **4** |
| **13** | 1335.7995 | 668.4034 | 1318.7729 | 659.8901 | 1317.7889 | 659.3981 | **K** | 274.2238 | 137.6155 | 257.1972 | 129.1022 |  |  | **3** |
| **14** | 1463.8944 | 732.4509 | 1446.8679 | 723.9376 | 1445.8839 | 723.4456 | **K** | 146.1288 | 73.5680 | 129.1022 | 65.0548 |  |  | **2** |
| **15** |  |  |  |  |  |  | **G** | 18.0338 | 9.5206 |  |  |  |  | **1** |

8. XPF-St6

|  | **Observed Mr** | **Expected Mr** | **Calculated Mr** | **Delta** | **Score** | **Peptide** |
| --- | --- | --- | --- | --- | --- | --- |
| **XPF-St6** | 857.2650 | 2568.7733 | 2568.4792 | 0.2941 | 49 | R.GVWSTILGGLKKFAKGGLDAIVNPK.R |


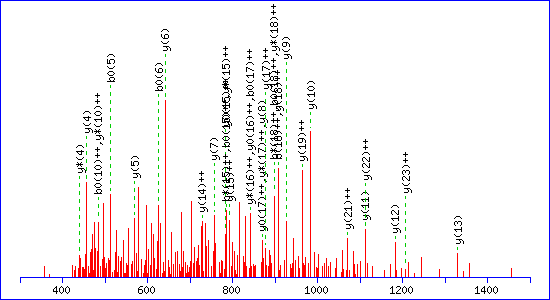


| **#** | **b** | **b^++^** | **b*** | **b*^++^** | **b^0^** | **b^0++^** | **Seq.** | **y** | **y^++^** | **y*** | **y*^++^** | **y^0^** | **y^0++^** | **#** |
| --- | --- | --- | --- | --- | --- | --- | --- | --- | --- | --- | --- | --- | --- | --- |
| **1** | 58.0287 | 29.5180 |  |  |  |  | **G** |  |  |  |  |  |  | **25** |
| **2** | 157.0972 | 79.0522 |  |  |  |  | **V** | 2512.4650 | 1256.7361 | 2495.4384 | 1248.2229 | 2494.4544 | 1247.7309 | **24** |
| **3** | 343.1765 | 172.0919 |  |  |  |  | **W** | 2413.3966 | **1207.2019** | 2396.3700 | 1198.6887 | 2395.3860 | 1198.1966 | **23** |
| **4** | 430.2085 | 215.6079 |  |  | 412.1979 | 206.6026 | **S** | 2227.3173 | **1114.1623** | 2210.2907 | 1105.6490 | 2209.3067 | 1105.1570 | **22** |
| **5** | 531.2562 | 266.1317 |  |  | **513.2456** | 257.1264 | **T** | 2140.2852 | **1070.6463** | 2123.2587 | 1062.1330 | 2122.2747 | 1061.6410 | **21** |
| **6** | 644.3402 | 322.6738 |  |  | **626.3297** | 313.6685 | **I** | 2039.2376 | 1020.1224 | 2022.2110 | 1011.6091 | 2021.2270 | 1011.1171 | **20** |
| **7** | 757.4243 | 379.2158 |  |  | 739.4137 | 370.2105 | **L** | 1926.1535 | **963.5804** | 1909.1269 | 955.0671 | 1908.1429 | 954.5751 | **19** |
| **8** | 814.4458 | 407.7265 |  |  | 796.4352 | 398.7212 | **G** | 1813.0694 | **907.0384** | 1796.0429 | **898.5251** | 1795.0589 | 898.0331 | **18** |
| **9** | 871.4672 | 436.2373 |  |  | 853.4567 | 427.2320 | **G** | 1756.0480 | **878.5276** | 1739.0214 | **870.0143** | 1738.0374 | **869.5223** | **17** |
| **10** | 984.5513 | 492.7793 |  |  | 966.5407 | **483.7740** | **L** | 1699.0265 | 850.0169 | 1682.0000 | **841.5036** | 1681.0159 | **841.0116** | **16** |
| **11** | 1112.6463 | 556.8268 | 1095.6197 | 548.3135 | 1094.6357 | 547.8215 | **K** | 1585.9424 | **793.4749** | 1568.9159 | **784.9616** | 1567.9319 | **784.4696** | **15** |
| **12** | 1240.7412 | 620.8742 | 1223.7147 | 612.3610 | 1222.7307 | 611.8690 | **K** | 1457.8475 | **729.4274** | 1440.8209 | 720.9141 | 1439.8369 | 720.4221 | **14** |
| **13** | 1387.8096 | 694.4085 | 1370.7831 | 685.8952 | 1369.7991 | 685.4032 | **F** | **1329.7525** | 665.3799 | 1312.7260 | 656.8666 | 1311.7419 | 656.3746 | **13** |
| **14** | 1458.8467 | 729.9270 | 1441.8202 | 721.4137 | 1440.8362 | 720.9217 | **A** | **1182.6841** | 591.8457 | 1165.6575 | 583.3324 | 1164.6735 | 582.8404 | **12** |
| **15** | 1586.9417 | 793.9745 | 1569.9152 | **785.4612** | 1568.9311 | **784.9692** | **K** | **1111.6470** | 556.3271 | 1094.6204 | 547.8139 | 1093.6364 | 547.3218 | **11** |
| **16** | 1643.9632 | 822.4852 | 1626.9366 | 813.9720 | 1625.9526 | 813.4799 | **G** | **983.5520** | 492.2796 | 966.5255 | **483.7664** | 965.5415 | 483.2744 | **10** |
| **17** | 1700.9846 | 850.9960 | 1683.9581 | 842.4827 | 1682.9741 | **841.9907** | **G** | **926.5306** | 463.7689 | 909.5040 | 455.2556 | 908.5200 | 454.7636 | **9** |
| **18** | 1814.0687 | **907.5380** | 1797.0422 | **899.0247** | 1796.0581 | **898.5327** | **L** | **869.5091** | 435.2582 | 852.4825 | 426.7449 | 851.4985 | 426.2529 | **8** |
| **19** | 1929.0956 | 965.0515 | 1912.0691 | 956.5382 | 1911.0851 | 956.0462 | **D** | **756.4250** | 378.7162 | 739.3985 | 370.2029 | 738.4145 | 369.7109 | **7** |
| **20** | 2000.1328 | 1000.5700 | 1983.1062 | 992.0567 | 1982.1222 | 991.5647 | **A** | **641.3981** | 321.2027 | 624.3715 | 312.6894 |  |  | **6** |
| **21** | 2113.2168 | 1057.1120 | 2096.1903 | 1048.5988 | 2095.2063 | 1048.1068 | **I** | **570.3610** | 285.6841 | 553.3344 | 277.1708 |  |  | **5** |
| **22** | 2212.2852 | 1106.6463 | 2195.2587 | 1098.1330 | 2194.2747 | 1097.6410 | **V** | **457.2769** | 229.1421 | **440.2504** | 220.6288 |  |  | **4** |
| **23** | 2326.3282 | 1163.6677 | 2309.3016 | 1155.1544 | 2308.3176 | 1154.6624 | **N** | 358.2085 | 179.6079 | 341.1819 | 171.0946 |  |  | **3** |
| **24** | 2423.3809 | 1212.1941 | 2406.3544 | 1203.6808 | 2405.3704 | 1203.1888 | **P** | 244.1656 | 122.5864 | 227.1390 | 114.0731 |  |  | **2** |
| **25** |  |  |  |  |  |  | **K** | 147.1128 | 74.0600 | 130.0863 | 65.5468 |  |  | **1** |

9. XPF-St7

|  | **Observed Mr** | **Expected Mr** | **Calculated Mr** | **Delta** | **Score** | **Peptide** |
| --- | --- | --- | --- | --- | --- | --- |
| **XPF-St7** | 1254.8825 | 2507.7504 | 2507.4588 | 0.2917 | 85 | R.GLLSNVAGLLKQFAKGGVNAVLNPK.R |


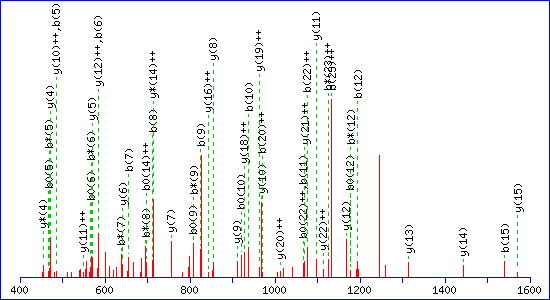


| **#** | **b** | **b^++^** | **b*** | **b*^++^** | **b^0^** | **b^0++^** | **Seq.** | **y** | **y^++^** | **y*** | **y*^++^** | **y^0^** | **y^0++^** | **#** |
| --- | --- | --- | --- | --- | --- | --- | --- | --- | --- | --- | --- | --- | --- | --- |
| **1** | 58.0287 | 29.5180 |  |  |  |  | **G** |  |  |  |  |  |  | **25** |
| **2** | 171.1128 | 86.0600 |  |  |  |  | **L** | 2451.4446 | 1226.2259 | 2434.4180 | 1217.7127 | 2433.4340 | 1217.2207 | **24** |
| **3** | 284.1969 | 142.6021 |  |  |  |  | **L** | 2338.3605 | 1169.6839 | 2321.3340 | 1161.1706 | 2320.3500 | 1160.6786 | **23** |
| **4** | 371.2289 | 186.1181 |  |  | 353.2183 | 177.1128 | **S** | 2225.2765 | **1113.1419** | 2208.2499 | 1104.6286 | 2207.2659 | 1104.1366 | **22** |
| **5** | **485.2718** | 243.1395 | **468.2453** | 234.6263 | **467.2613** | 234.1343 | **N** | 2138.2444 | **1069.6259** | 2121.2179 | 1061.1126 |  |  | **21** |
| **6** | **584.3402** | 292.6738 | **567.3137** | 284.1605 | **566.3297** | 283.6685 | **V** | 2024.2015 | **1012.6044** | 2007.1750 | 1004.0911 |  |  | **20** |
| **7** | **655.3774** | 328.1923 | **638.3508** | 319.6790 | 637.3668 | 319.1870 | **A** | 1925.1331 | **963.0702** | 1908.1065 | 954.5569 |  |  | **19** |
| **8** | **712.3988** | 356.7030 | **695.3723** | 348.1898 | 694.3883 | 347.6978 | **G** | 1854.0960 | **927.5516** | 1837.0694 | 919.0384 |  |  | **18** |
| **9** | **825.4829** | 413.2451 | **808.4563** | 404.7318 | **807.4723** | 404.2398 | **L** | 1797.0745 | 899.0409 | 1780.0480 | 890.5276 |  |  | **17** |
| **10** | **938.5669** | 469.7871 | 921.5404 | 461.2738 | **920.5564** | 460.7818 | **L** | 1683.9905 | **842.4989** | 1666.9639 | 833.9856 |  |  | **16** |
| **11** | **1066.6619** | 533.8346 | 1049.6354 | 525.3213 | 1048.6513 | 524.8293 | **K** | **1570.9064** | 785.9568 | 1553.8798 | 777.4436 |  |  | **15** |
| **12** | **1194.7205** | 597.8639 | **1177.6939** | 589.3506 | **1176.7099** | 588.8586 | **Q** | **1442.8114** | 721.9094 | 1425.7849 | **713.3961** |  |  | **14** |
| **13** | 1341.7889 | 671.3981 | 1324.7623 | 662.8848 | 1323.7783 | 662.3928 | **F** | **1314.7528** | 657.8801 | 1297.7263 | 649.3668 |  |  | **13** |
| **14** | 1412.8260 | 706.9166 | 1395.7995 | 698.4034 | 1394.8154 | **697.9114** | **A** | **1167.6844** | **584.3459** | 1150.6579 | 575.8326 |  |  | **12** |
| **15** | **1540.9210** | 770.9641 | 1523.8944 | 762.4509 | 1522.9104 | 761.9588 | **K** | **1096.6473** | **548.8273** | 1079.6208 | 540.3140 |  |  | **11** |
| **16** | 1597.9424 | 799.4749 | 1580.9159 | 790.9616 | 1579.9319 | 790.4696 | **G** | **968.5524** | **484.7798** | 951.5258 | 476.2665 |  |  | **10** |
| **17** | 1654.9639 | 827.9856 | 1637.9374 | 819.4723 | 1636.9533 | 818.9803 | **G** | **911.5309** | 456.2691 | 894.5043 | 447.7558 |  |  | **9** |
| **18** | 1754.0323 | 877.5198 | 1737.0058 | 869.0065 | 1736.0218 | 868.5145 | **V** | **854.5094** | 427.7584 | 837.4829 | 419.2451 |  |  | **8** |
| **19** | 1868.0752 | 934.5413 | 1851.0487 | 926.0280 | 1850.0647 | 925.5360 | **N** | **755.4410** | 378.2241 | 738.4145 | 369.7109 |  |  | **7** |
| **20** | 1939.1124 | **970.0598** | 1922.0858 | 961.5465 | 1921.1018 | 961.0545 | **A** | **641.3981** | 321.2027 | 624.3715 | 312.6894 |  |  | **6** |
| **21** | 2038.1808 | 1019.5940 | 2021.1542 | 1011.0807 | 2020.1702 | 1010.5887 | **V** | **570.3610** | 285.6841 | 553.3344 | 277.1709 |  |  | **5** |
| **22** | 2151.2648 | **1076.1361** | 2134.2383 | 1067.6228 | 2133.2543 | **1067.1308** | **L** | **471.2926** | 236.1499 | **454.2660** | 227.6366 |  |  | **4** |
| **23** | 2265.3078 | **1133.1575** | 2248.2812 | **1124.6442** | 2247.2972 | 1124.1522 | **N** | 358.2085 | 179.6079 | 341.1819 | 171.0946 |  |  | **3** |
| **24** | 2362.3605 | 1181.6839 | 2345.3340 | 1173.1706 | 2344.3500 | 1172.6786 | **P** | 244.1656 | 122.5864 | 227.1390 | 114.0731 |  |  | **2** |
| **25** |  |  |  |  |  |  | **K** | 147.1128 | 74.0600 | 130.0863 | 65.5468 |  |  | **1** |

10. PFQa-St1

|  | **Observed Mr** | **Expected Mr** | **Calculated Mr** | **Delta** | **Score** | **Peptide** |
| --- | --- | --- | --- | --- | --- | --- |
| **PFQa-St1** | 741.0672 | 1480.1199 | 1479.9541 | 0.1658 | 3 | R.FIGALLGPLLNLLKa.(G)R |


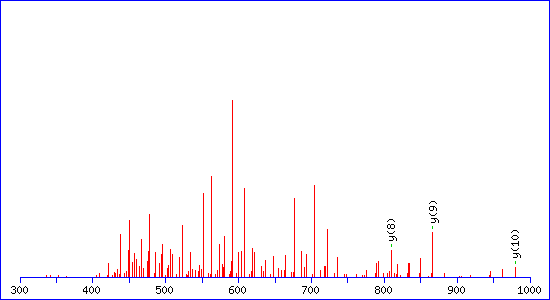


| **#** | **b** | **b^++^** | **b*** | **b*^++^** | **Seq.** | **y** | **y^++^** | **y*** | **y*^++^** | **#** |
| --- | --- | --- | --- | --- | --- | --- | --- | --- | --- | --- |
| **1** | 148.0757 | 74.5415 |  |  | **F** |  |  |  |  | **15** |
| **2** | 261.1598 | 131.0835 |  |  | **I** | 1333.8930 | 667.4501 | 1316.8664 | 658.9368 | **14** |
| **3** | 318.1812 | 159.5942 |  |  | **G** | 1220.8089 | 610.9081 | 1203.7824 | 602.3948 | **13** |
| **4** | 389.2183 | 195.1128 |  |  | **A** | 1163.7874 | 582.3974 | 1146.7609 | 573.8841 | **12** |
| **5** | 502.3024 | 251.6548 |  |  | **L** | 1092.7503 | 546.8788 | 1075.7238 | 538.3655 | **11** |
| **6** | 615.3865 | 308.1969 |  |  | **L** | **979.6663** | 490.3368 | 962.6397 | 481.8235 | **10** |
| **7** | 672.4079 | 336.7076 |  |  | **G** | **866.5822** | 433.7947 | 849.5557 | 425.2815 | **9** |
| **8** | 769.4607 | 385.2340 |  |  | **P** | **809.5607** | 405.2840 | 792.5342 | 396.7707 | **8** |
| **9** | 882.5448 | 441.7760 |  |  | **L** | 712.5080 | 356.7576 | 695.4814 | 348.2443 | **7** |
| **10** | 995.6288 | 498.3180 |  |  | **L** | 599.4239 | 300.2156 | 582.3974 | 291.7023 | **6** |
| **11** | 1109.6717 | 555.3395 | 1092.6452 | 546.8262 | **N** | 486.3398 | 243.6736 | 469.3133 | 235.1603 | **5** |
| **12** | 1222.7558 | 611.8815 | 1205.7293 | 603.3683 | **L** | 372.2969 | 186.6521 | 355.2704 | 178.1388 | **4** |
| **13** | 1335.8399 | 668.4236 | 1318.8133 | 659.9103 | **L** | 259.2129 | 130.1101 | 242.1863 | 121.5968 | **3** |
| **14** | 1463.9348 | 732.4711 | 1446.9083 | 723.9578 | **K** | 146.1288 | 73.5680 | 129.1022 | 65.0548 | **2** |
| **15** |  |  |  |  | **G** | 18.0338 | 9.5206 |  |  | **1** |

11. PGLa-St2

|  | **Observed Mr** | **Expected Mr** | **Calculated Mr** | **Delta** | **Score** | **Peptide** |
| --- | --- | --- | --- | --- | --- | --- |
| **PGLa-St2** | 1009.6862 | 2017.3579 | 2017.1506 | 0.2073 | 96 | R.GMATKAGTAFGKAAKAIIGAALa.(G)R |


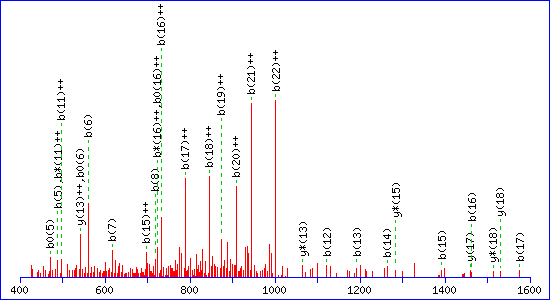


| **#** | **b** | **b^++^** | **b*** | **b*^++^** | **b^0^** | **b^0++^** | **Seq.** | **y** | **y^++^** | **y*** | **y*^++^** | **y^0^** | **y^0++^** | **#** |
| --- | --- | --- | --- | --- | --- | --- | --- | --- | --- | --- | --- | --- | --- | --- |
| **1** | 58.0287 | 29.5180 |  |  |  |  | **G** |  |  |  |  |  |  | **23** |
| **2** | 189.0692 | 95.0383 |  |  |  |  | **M** | 1961.1365 | 981.0719 | 1944.1099 | 972.5586 | 1943.1259 | 972.0666 | **22** |
| **3** | 260.1063 | 130.5568 |  |  |  |  | **A** | 1830.0960 | 915.5516 | 1813.0694 | 907.0384 | 1812.0854 | 906.5463 | **21** |
| **4** | 361.1540 | 181.0806 |  |  | 343.1435 | 172.0754 | **T** | 1759.0589 | 880.0331 | 1742.0323 | 871.5198 | 1741.0483 | 871.0278 | **20** |
| **5** | **489.2490** | 245.1281 | 472.2224 | 236.6149 | **471.2384** | 236.1228 | **K** | 1658.0112 | 829.5092 | 1640.9846 | 820.9960 | 1640.0006 | 820.5040 | **19** |
| **6** | **560.2861** | 280.6467 | 543.2595 | 272.1334 | **542.2755** | 271.6414 | **A** | **1529.9162** | 765.4618 | **1512.8897** | 756.9485 | 1511.9057 | 756.4565 | **18** |
| **7** | **617.3076** | 309.1574 | 600.2810 | 300.6441 | 599.2970 | 300.1521 | **G** | **1458.8791** | 729.9432 | 1441.8526 | 721.4299 | 1440.8685 | 720.9379 | **17** |
| **8** | **718.3552** | 359.6813 | 701.3287 | 351.1680 | 700.3447 | 350.6760 | **T** | 1401.8576 | 701.4325 | 1384.8311 | 692.9192 | 1383.8471 | 692.4272 | **16** |
| **9** | 789.3924 | 395.1998 | 772.3658 | 386.6865 | 771.3818 | 386.1945 | **A** | 1300.8100 | 650.9086 | **1283.7834** | 642.3953 |  |  | **15** |
| **10** | 936.4608 | 468.7340 | 919.4342 | 460.2207 | 918.4502 | 459.7287 | **F** | 1229.7729 | 615.3901 | 1212.7463 | 606.8768 |  |  | **14** |
| **11** | 993.4822 | **497.2448** | 976.4557 | **488.7315** | 975.4717 | 488.2395 | **G** | 1082.7044 | **541.8559** | **1065.6779** | 533.3426 |  |  | **13** |
| **12** | **1121.5772** | 561.2922 | 1104.5506 | 552.7790 | 1103.5666 | 552.2870 | **K** | 1025.6830 | 513.3451 | 1008.6564 | 504.8319 |  |  | **12** |
| **13** | **1192.6143** | 596.8108 | 1175.5878 | 588.2975 | 1174.6037 | 587.8055 | **A** | 897.5880 | 449.2976 | 880.5615 | 440.7844 |  |  | **11** |
| **14** | **1263.6514** | 632.3293 | 1246.6249 | 623.8161 | 1245.6409 | 623.3241 | **A** | 826.5509 | 413.7791 | 809.5244 | 405.2658 |  |  | **10** |
| **15** | **1391.7464** | **696.3768** | 1374.7198 | 687.8636 | 1373.7358 | 687.3715 | **K** | 755.5138 | 378.2605 | 738.4872 | 369.7473 |  |  | **9** |
| **16** | **1462.7835** | **731.8954** | 1445.7569 | **723.3821** | 1444.7729 | **722.8901** | **A** | 627.4188 | 314.2131 |  |  |  |  | **8** |
| **17** | **1575.8676** | **788.4374** | 1558.8410 | 779.9241 | 1557.8570 | 779.4321 | **I** | 556.3817 | 278.6945 |  |  |  |  | **7** |
| **18** | 1688.9516 | **844.9795** | 1671.9251 | 836.4662 | 1670.9411 | 835.9742 | **I** | 443.2976 | 222.1525 |  |  |  |  | **6** |
| **19** | 1745.9731 | **873.4902** | 1728.9465 | 864.9769 | 1727.9625 | 864.4849 | **G** | 330.2136 | 165.6104 |  |  |  |  | **5** |
| **20** | 1817.0102 | **909.0087** | 1799.9837 | 900.4955 | 1798.9996 | 900.0035 | **A** | 273.1921 | 137.0997 |  |  |  |  | **4** |
| **21** | 1888.0473 | **944.5273** | 1871.0208 | 936.0140 | 1870.0368 | 935.5220 | **A** | 202.1550 | 101.5811 |  |  |  |  | **3** |
| **22** | 2001.1314 | **1001.0693** | 1984.1048 | 992.5561 | 1983.1208 | 992.0640 | **L** | 131.1179 | 66.0626 |  |  |  |  | **2** |
| **23** |  |  |  |  |  |  | **G** | 18.0338 | 9.5206 |  |  |  |  | **1** |

12. PGLa-St3

|  | **Observed Mr** | **Expected Mr** | **Calculated Mr** | **Delta** | **Score** | **Peptide** |
| --- | --- | --- | --- | --- | --- | --- |
| **PGLa-St3** | 1035.7401 | 2069.4655 | 2069.2395 | 0.2261 | 104 | R.GMATKAGTVLGKVTKAIIGAALa.(G)R |


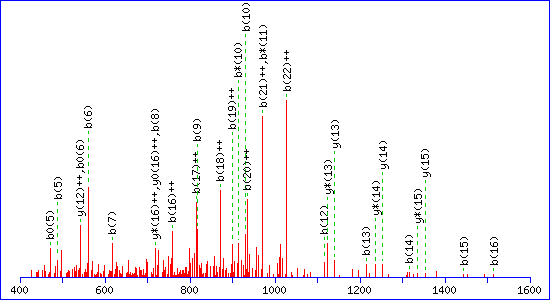


| **#** | **b** | **b^++^** | **b*** | **b*^++^** | **b^0^** | **b^0++^** | **Seq.** | **y** | **y^++^** | **y*** | **y*^++^** | **y^0^** | **y^0++^** | **#** |
| --- | --- | --- | --- | --- | --- | --- | --- | --- | --- | --- | --- | --- | --- | --- |
| **1** | 58.0287 | 29.5180 |  |  |  |  | **G** |  |  |  |  |  |  | **23** |
| **2** | 189.0692 | 95.0383 |  |  |  |  | **M** | 2013.2253 | 1007.1163 | 1996.1987 | 998.6030 | 1995.2147 | 998.1110 | **22** |
| **3** | 260.1063 | 130.5568 |  |  |  |  | **A** | 1882.1848 | 941.5960 | 1865.1583 | 933.0828 | 1864.1742 | 932.5908 | **21** |
| **4** | 361.1540 | 181.0806 |  |  | 343.1435 | 172.0754 | **T** | 1811.1477 | 906.0775 | 1794.1211 | 897.5642 | 1793.1371 | 897.0722 | **20** |
| **5** | **489.2490** | 245.1281 | 472.2224 | 236.6149 | **471.2384** | 236.1228 | **K** | 1710.1000 | 855.5536 | 1693.0735 | 847.0404 | 1692.0894 | 846.5484 | **19** |
| **6** | **560.2861** | 280.6467 | 543.2595 | 272.1334 | **542.2755** | 271.6414 | **A** | 1582.0050 | 791.5062 | 1564.9785 | 782.9929 | 1563.9945 | 782.5009 | **18** |
| **7** | **617.3076** | 309.1574 | 600.2810 | 300.6441 | 599.2970 | 300.1521 | **G** | 1510.9679 | 755.9876 | 1493.9414 | 747.4743 | 1492.9574 | 746.9823 | **17** |
| **8** | **718.3552** | 359.6813 | 701.3287 | 351.1680 | 700.3447 | 350.6760 | **T** | 1453.9465 | 727.4769 | 1436.9199 | **718.9636** | 1435.9359 | **718.4716** | **16** |
| **9** | **817.4237** | 409.2155 | 800.3971 | 400.7022 | 799.4131 | 400.2102 | **V** | **1352.8988** | 676.9530 | **1335.8722** | 668.4398 | 1334.8882 | 667.9477 | **15** |
| **10** | **930.5077** | 465.7575 | **913.4812** | 457.2442 | 912.4972 | 456.7522 | **L** | **1253.8304** | 627.4188 | **1236.8038** | 618.9055 | 1235.8198 | 618.4135 | **14** |
| **11** | 987.5292 | 494.2682 | **970.5026** | 485.7550 | 969.5186 | 485.2629 | **G** | **1140.7463** | 570.8768 | **1123.7198** | 562.3635 | 1122.7357 | 561.8715 | **13** |
| **12** | **1115.6241** | 558.3157 | 1098.5976 | 549.8024 | 1097.6136 | 549.3104 | **K** | 1083.7248 | **542.3661** | 1066.6983 | 533.8528 | 1065.7143 | 533.3608 | **12** |
| **13** | **1214.6926** | 607.8499 | 1197.6660 | 599.3366 | 1196.6820 | 598.8446 | **V** | 955.6299 | 478.3186 | 938.6033 | 469.8053 | 937.6193 | 469.3133 | **11** |
| **14** | **1315.7402** | 658.3738 | 1298.7137 | 649.8605 | 1297.7297 | 649.3685 | **T** | 856.5615 | 428.7844 | 839.5349 | 420.2711 | 838.5509 | 419.7791 | **10** |
| **15** | **1443.8352** | 722.4212 | 1426.8087 | 713.9080 | 1425.8246 | 713.4160 | **K** | 755.5138 | 378.2605 | 738.4872 | 369.7473 |  |  | **9** |
| **16** | **1514.8723** | **757.9398** | 1497.8458 | 749.4265 | 1496.8617 | 748.9345 | **A** | 627.4188 | 314.2131 |  |  |  |  | **8** |
| **17** | 1627.9564 | **814.4818** | 1610.9298 | 805.9686 | 1609.9458 | 805.4765 | **I** | 556.3817 | 278.6945 |  |  |  |  | **7** |
| **18** | 1741.0404 | **871.0239** | 1724.0139 | 862.5106 | 1723.0299 | 862.0186 | **I** | 443.2976 | 222.1525 |  |  |  |  | **6** |
| **19** | 1798.0619 | **899.5346** | 1781.0354 | 891.0213 | 1780.0513 | 890.5293 | **G** | 330.2136 | 165.6104 |  |  |  |  | **5** |
| **20** | 1869.0990 | **935.0531** | 1852.0725 | 926.5399 | 1851.0885 | 926.0479 | **A** | 273.1921 | 137.0997 |  |  |  |  | **4** |
| **21** | 1940.1361 | **970.5717** | 1923.1096 | 962.0584 | 1922.1256 | 961.5664 | **A** | 202.1550 | 101.5811 |  |  |  |  | **3** |
| **22** | 2053.2202 | **1027.1137** | 2036.1936 | 1018.6005 | 2035.2096 | 1018.1085 | **L** | 131.1179 | 66.0626 |  |  |  |  | **2** |
| **23** |  |  |  |  |  |  | **G** | 18.0338 | 9.5206 |  |  |  |  | **1** |

13. PLD-St1

|  | **Observed Mr** | **Expected Mr** | **Calculated Mr** | **Delta** | **Score** | **Peptide** |
| --- | --- | --- | --- | --- | --- | --- |
| **PLD-St1** | 850.6018 | 1699.1890 | 1698.9920 | 0.1970 | 27 | A.LLDLEKKIKQFLPD.* |


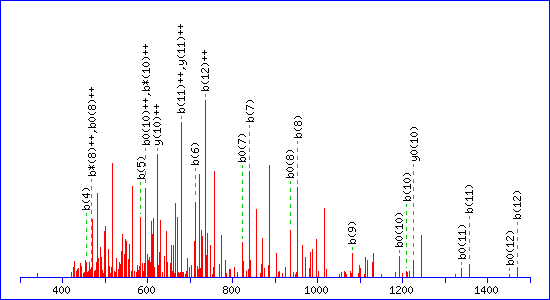


| **#** | **b** | **b^++^** | **b*** | **b*^++^** | **b^0^** | **b^0++^** | **Seq.** | **y** | **y^++^** | **y*** | **y*^++^** | **y^0^** | **y^0++^** | **#** |
| --- | --- | --- | --- | --- | --- | --- | --- | --- | --- | --- | --- | --- | --- | --- |
| **1** | 114.0913 | 57.5493 |  |  |  |  | **L** |  |  |  |  |  |  | **14** |
| **2** | 227.1754 | 114.0913 |  |  |  |  | **L** | 1585.9312 | 793.4692 | 1568.9046 | 784.9560 | 1567.9206 | 784.4640 | **13** |
| **3** | 342.2023 | 171.6048 |  |  | 324.1918 | 162.5995 | **D** | 1472.8471 | 736.9272 | 1455.8206 | 728.4139 | 1454.8366 | 727.9219 | **12** |
| **4** | **455.2864** | 228.1468 |  |  | 437.2758 | 219.1416 | **L** | 1357.8202 | **679.4137** | 1340.7936 | 670.9005 | 1339.8096 | 670.4085 | **11** |
| **5** | **584.3290** | 292.6681 |  |  | 566.3184 | 283.6629 | **E** | 1244.7361 | **622.8717** | 1227.7096 | 614.3584 | **1226.7256** | 613.8664 | **10** |
| **6** | **712.4240** | 356.7156 | 695.3974 | 348.2023 | 694.4134 | 347.7103 | **K** | 1115.6935 | 558.3504 | 1098.6670 | 549.8371 | 1097.6830 | 549.3451 | **9** |
| **7** | **840.5189** | 420.7631 | 823.4924 | 412.2498 | **822.5084** | 411.7578 | **K** | 987.5986 | 494.3029 | 970.5720 | 485.7896 | 969.5880 | 485.2976 | **8** |
| **8** | **953.6030** | 477.3051 | 936.5764 | **468.7919** | **935.5924** | **468.2999** | **I** | 859.5036 | 430.2554 | 842.4771 | 421.7422 | 841.4930 | 421.2502 | **7** |
| **9** | **1081.6980** | 541.3526 | 1064.6714 | 532.8393 | 1063.6874 | 532.3473 | **K** | 746.4195 | 373.7134 | 729.3930 | 365.2001 | 728.4090 | 364.7081 | **6** |
| **10** | **1209.7565** | 605.3819 | 1192.7300 | **596.8686** | **1191.7460** | **596.3766** | **Q** | 618.3246 | 309.6659 | 601.2980 | 301.1527 | 600.3140 | 300.6606 | **5** |
| **11** | **1356.8249** | **678.9161** | 1339.7984 | 670.4028 | **1338.8144** | 669.9108 | **F** | 490.2660 | 245.6366 |  |  | 472.2554 | 236.6314 | **4** |
| **12** | **1469.9090** | **735.4581** | 1452.8825 | 726.9449 | **1451.8984** | 726.4529 | **L** | 343.1976 | 172.1024 |  |  | 325.1870 | 163.0971 | **3** |
| **13** | 1566.9618 | 783.9845 | 1549.9352 | 775.4713 | 1548.9512 | 774.9792 | **P** | 230.1135 | 115.5604 |  |  | 212.1030 | 106.5551 | **2** |
| **14** |  |  |  |  |  |  | **D** | 133.0608 | 67.0340 |  |  | 115.0502 | 58.0287 | **1** |
